# Destination shapes antibiotic resistance gene acquisitions, abundance increases, and diversity changes in Dutch travelers

**DOI:** 10.1186/s13073-021-00893-z

**Published:** 2021-06-07

**Authors:** Alaric W. D’Souza, Manish Boolchandani, Sanket Patel, Gianluca Galazzo, Jarne M. van Hattem, Maris S. Arcilla, Damian C. Melles, Menno D. de Jong, Constance Schultsz, COMBAT Consortium, Martin C. J. Bootsma, Perry J. van Genderen, Abraham Goorhuis, Martin P. Grobusch, Nicky Molhoek, Astrid M. L. Oude Lashof, Ellen E. Stobberingh, Henri A. Verbrugh, Gautam Dantas, John Penders

**Affiliations:** 1 The Edison Family Center for Genome Sciences and Systems Biology, Washington University School of Medicine, St. Louis, MO USA; 2 Department of Pathology and Immunology, Washington University School of Medicine, St. Louis, MO USA; 3 Department of Medical Microbiology, Care and Public Health Research Institute (CAPHRI), Maastricht University Medical Center, Maastricht, The Netherlands; 4 Department of Medical Microbiology, Amsterdam University Medical Center, Location AMC, Amsterdam, The Netherlands; 5 Department of Medical Microbiology and Infectious Diseases, Erasmus University Medical Centre, Rotterdam, The Netherlands; 6 Department of Global Health, Amsterdam Institute for Global Health and Development, AMC, Amsterdam, The Netherlands; 7 Department of Molecular Microbiology, Washington University School of Medicine, St. Louis, MO USA; 8 Department of Biomedical Engineering, Washington University in St. Louis, St. Louis, MO USA; 9 School for Nutrition and Translational Research in Metabolism (NUTRIM), Maastricht University Medical Center, Maastricht, The Netherlands

**Keywords:** Resistome, Antibiotic resistance, Travel, mcr-1, β-Lactamases, ESBL

## Abstract

**Background:**

Antimicrobial-resistant bacteria and their antimicrobial resistance (AMR) genes can spread by hitchhiking in human guts. International travel can exacerbate this public health threat when travelers acquire AMR genes endemic to their destinations and bring them back to their home countries. Prior studies have demonstrated travel-related acquisition of specific opportunistic pathogens and AMR genes, but the extent and magnitude of travel’s effects on the gut resistome remain largely unknown.

**Methods:**

Using whole metagenomic shotgun sequencing, functional metagenomics, and Dirichlet multinomial mixture models, we investigated the abundance, diversity, function, resistome architecture, and context of AMR genes in the fecal microbiomes of 190 Dutch individuals, before and after travel to diverse international locations.

**Results:**

Travel markedly increased the abundance and α-diversity of AMR genes in the travelers’ gut resistome, and we determined that 56 unique AMR genes showed significant acquisition following international travel. These acquisition events were biased towards AMR genes with efflux, inactivation, and target replacement resistance mechanisms. Travel-induced shaping of the gut resistome had distinct correlations with geographical destination, so individuals returning to The Netherlands from the same destination country were more likely to have similar resistome features. Finally, we identified and detailed specific acquisition events of high-risk, mobile genetic element-associated AMR genes including *qnr* fluoroquinolone resistance genes, *bla*_CTX-M_ family extended-spectrum β-lactamases, and the plasmid-borne *mcr-1* colistin resistance gene.

**Conclusions:**

Our results show that travel shapes the architecture of the human gut resistome and results in AMR gene acquisition against a variety of antimicrobial drug classes. These broad acquisitions highlight the putative risks that international travel poses to public health by gut resistome perturbation and the global spread of locally endemic AMR genes.

**Supplementary Information:**

The online version contains supplementary material available at 10.1186/s13073-021-00893-z.

## Background

Antimicrobial resistance (AMR) is a major global public health threat with a high mortality cost [1–6]. AMR bacterial infections now frequently render antibiotics ineffective and limit clinicians’ antibiotic treatment options. This trend threatens 70 years of progress in treating bacterial infectious diseases.

AMR is rising worldwide, but there are large geographic differences in the prevalence and type of resistant bacteria and their AMR genes [7, 8]. Low- and middle-income countries generally have higher endemic AMR than high-income countries, mainly driven by antibiotic overuse in humans and animals [6–10]. International travel can facilitate the transfer of resistant bacteria and AMR genes from their endemic regions to other locations around the globe [11–18].

An AMR gene’s ability to spread via international travel is context-dependent [11, 19, 20]. Context includes the AMR gene’s prevalence in the endemic region, the specific bacteria harboring the AMR gene, and the other genetic elements colocalized with the gene. AMR genes such as extended-spectrum β-lactamases (ESBLs), *qnr*, and *mcr-1* are often associated with mobile genetic elements like plasmids and are of particularly high concern due to their ease of spread [8, 11, 21–23].

Returning travelers are rarely tested for resistant bacteria or AMR genes unless they manifest clinical symptoms, so the magnitude of AMR gene acquisition risk from international travel remains underdetermined. Using microbial culture, studies have shown significant acquisition rates of opportunistic pathogens, such as ESBL-producing Enterobacteriaceae [16, 18, 24, 25]. These studies identified specific pathogenic bacteria acquired during international travel, and several identified specific AMR genes acquired during travel [16, 26, 27]. But the effect of international travel on AMR is most likely not limited to opportunistic pathogens such as *Escherichia coli* or to ESBL-encoding resistance genes. A broader risk assessment must include acquired commensals and their potential horizontal transfer of AMR genes with host microbiomes.

Rapid advancements in sequencing technology, bioinformatics, and database curation facilitate quantitative insight into the human microbiome’s role as an AMR reservoir in a broader context and how this role might be influenced by international travel [8, 28]. We can sequence all extracted DNA using shotgun metagenomic sequencing [8, 28, 29], and we can directly identify AMR genes in these shotgun metagenomes by mapping reads to curated AMR gene databases [8, 28]. Though AMR gene databases and identification techniques have made significant advancements in recent years, they still rely heavily on the traditional microbiological culture that excludes many bacteria [28]. Functional metagenomics is a powerful complementary method to more broadly survey AMR determinants without relying on culturing resistant bacteria [8, 28, 30]. Instead, functional metagenomics uses a cultivable indicator bacterium to identify functional AMR determinants from metagenomic samples via recombinant gene expression and phenotypic selection [30].

Here, we combine next-generation sequencing, functional metagenomics, and statistical modeling to investigate the abundance, diversity, function, context, and acquisition of AMR genes in a group of international travelers. Our results demonstrate that international travel is a significant perturbation to the gut resistome and reveal destination-specific changes to travelers’ resistomes including AMR gene acquisitions against last resort antibiotics and AMR gene colocalization with mobile genetic elements. These findings further our understanding of the role of travelers as potential reservoirs and spreaders of AMR.

## Methods

### Study design, sample collection, and DNA extraction

Samples for this project were selected from a subset of the broader Carriage Of Multiresistant Bacteria After Travel (COMBAT) study [17, 31]. Within this multicenter longitudinal cohort study, travelers were recruited at the outpatient travel clinics run by the Academic Medical Center (Amsterdam, The Netherlands), Havenziekenhuis (Rotterdam, The Netherlands), and Maastricht University Medical Center/Public Health Service South Limburg (Maastricht, The Netherlands). Minors, incapacitated subjects, and subjects that traveled abroad for shorter than 1 week or longer than 3 months were excluded from the study. In total, 2001 travelers were included and provided with fecal swab kits that included instructions, a modified Carey-Blair transport medium with an associated swab (Fecal Swab®; Copan, Brescia, Italy), a questionnaire, and paid postage. Before leaving for and immediately after returning from travel, subjects took samples from their stool using the fecal swab kits and mailed them to the lab. The methods for sample collection are described in detail in Arcilla et al. [31] and Arcilla et al. [17].

For the purpose of the present study, we limited the selection to travelers to Southeastern Asia, South Asia, North Africa, and Eastern Africa to have sufficient numbers per subregion. Subregions are defined according to the United Nations regional grouping M49 standard [32]. Travelers were excluded if they visited multiple subregions or consumed antibiotics in the 3 months before travel. Selections were made by stratified random sampling using SPSS.

Metagenomic DNA was extracted from stool samples using repeated bead-beating (RBB) combined with column-based purification according to protocol Q (IHMS_SOP 06 V2 - http://www.microbiome-standards.org/index.php?id=253) of the International Human Microbiome Standards consortium [33]. Bead-beating was done using the FastPrep™ Instrument (MP Biomedicals, Santa Ana, CA, USA) with 0.1-mm zirconium-silica beads (BioSpec Products, Bartlesville, OK, USA) to homogenize feces. DNA was finally purified by adapting to the QIAamp DNA Stool Mini kit columns (Qiagen, Hilden, Germany). A Qubit® fluorometer dsDNA HS Assay (Invitrogen) was used to quantify extracted DNA, and this DNA was stored at −20°C.

Extracted metagenomic DNA was diluted to 0.5 ng/μL and prepared for sequencing with a Nextera DNA Library Prep Kit (Illumina) using a modified Nextera protocol [34]. Libraries were purified using the Agencourt AMPure XP system (Beckman Coulter) and quantified using the Quant-iT PicoGreen dsDNA assay (Invitrogen). For each sequencing lane, 10 nM of approximately 96 samples was pooled three independent times. These pools were quantified using the Qubit® dsDNA BR Assay and combined in an equimolar fashion. Samples were submitted for 2×150 bp paired-end sequencing on an Illumina NextSeq High-Output platform with a target sequencing depth of 5 million reads per sample.

### Sequence quality filtering

Trimmomatic v0.36 [35] was used to trim Nextera adapter sequences and to quality filter sequenced reads with the following parameters:

Adapter = Nextera

Illuminaclip = 2:30:10:1:TRUE

Leading = 10

Traling = 10

Sliding window =4:15

Min length = 60

Deconseq v0.4.3 was used to remove human read contamination [36].

### Functional metagenomics

We constructed, sequenced, and analyzed 21 small-insert (>0.7 kb) functional metagenomics libraries which were screened for antibiotic resistance with adaptations to our previously published protocols [30, 37–44]. The experimental protocol for creation and screening of functional metagenomics library is briefly described below:

#### Construction of functional metagenomics libraries

The metagenomic DNA (mgDNA) of 190 post-travel samples were divided based on four different travel regions, and up to ten random samples from each region were pooled together for the construction of each functional metagenomic library (Additional file 1: Fig. S1). The pooled mgDNA was fragmented by partial restriction digestion using *BamHI*, *BglII*, and *BstYI* (New England Biolabs (NEB)) restriction enzymes. First, 1 μg of mgDNA was mixed with 1–5 units of both *BamHI* and *BglII* (NEB) in a total volume of 90 μl. The digest was put at 37 °C in an Eppendorf incubator, and aliquots of 15 μl were withdrawn after 5, 10, and 15 min and collected in an Eppendorf tube, containing 270 μl of absolute ethanol and 9 μl of 3M sodium acetate (pH=8) on ice. After withdrawing the third aliquot, 1–5 units of restriction enzyme *BstYI* (NEB) was added to the remaining digest. The incubation was continued at 37 °C, while withdrawing 15 μl every 5 min and pooling with the first aliquots, on ice. The pooled sample was mixed by vortexing and incubated at −70 °C for 5–10 min. The DNA was pelleted by centrifugating for 10 min at full speed in an Eppendorf centrifuge and subsequently washed once with 200 μl of 80% ethanol. After drying, the DNA pellet was dissolved in 50 μl of sterile water.

For size selection and purification of the partially restriction digested mgDNA, the MagVigen™ DNA select Kit (NVigen Inc.) was used according to the manufacturer’s instructions to retain fragments >700 bp. Finally, the sample was eluted in 30 μl of sterile water, and DNA concentration was quantified in a Qubit™ fluorometer (Invitrogen).

Vector pZE21-MCS was linearized by digestion with restriction enzyme *BamHI* and dephosphorylated using alkaline phosphatase (FastAP Thermosensitive Alkaline Phosphatase; Thermo Scientific), according to the manufacturer’s instructions. Ligation was performed using 50 ng of linearized, dephosphorylated pZE21-MCS vector and 100–150 ng of fragmented, size-selected insert DNA, according to the suppliers’ instructions (DNA ligation kit LONG; TaKaRa). Ligation reaction was performed for at least 3 h at 16 °C.

Subsequently, the ligation mixture was precipitated by adding 5 μl of 3 M sodium acetate pH 8 and 150 μl of absolute ethanol. The solution was mixed and incubated for 10 min at −70 °C, followed by a spin at full speed for 10 min in an Eppendorf centrifuge. The resulting DNA pellet was washed twice with freshly prepared 80% ethanol, air-dried, and dissolved in 5 μl sterile water. On ice, 25 μl of electrocompetent *E. coli* cells (E.cloni® 10G; Lucigen) was added to the ligated plasmid DNA, and electroporation was done according to the supplier’s instructions (1-mm cuvette, 10 μF, 600 Ω, 1800 V). Immediately after transformation, 2 ml of LB medium was added to the cells, and the suspension was incubated for 1 hat 37 °C in an orbital shaker.

The library titers were determined by plating 0.1 μl and 0.01 μl of recovered cells onto Luria-Broth (LB) agar plates containing 50 μg/ml kanamycin as previously described [30].

The remainder of recovered cells were grown overnight in 50 ml of LB broth containing 50 μg/ml kanamycin (LB-Kan) in a shaker (library amplification).

The culture was then centrifuged and re-suspended in 15 ml LB-Kan broth containing 15% glycerol and stored at −80 °C for subsequent screening.

#### Functional screening of antibiotic resistance

Each metagenomic expression library was screened on Mueller-Hinton agar with 50 μg/ml kanamycin and one of the 15 antibiotics at concentration listed in Additional file 2: Table S9. Before plating each library on antibiotic-containing growth media, the concentration of each library was adjusted such that 100 μl of library freezer stock contains at least 10× the total number of unique clones as determined at the time of library creation. To adjust the concentration, the freezer stock solution was either diluted with MH-Kan or centrifuged and reconstituted again in the appropriate volume for plating. The antibiotic selection plates were incubated for 16–24 h at 37°C to allow the growth of antibiotic-resistant clones. Additionally, for each antibiotic selection, a negative control plate of *E. coli* (E.cloni® 10G; Lucigen) transformed with unmodified pZE21 (without metagenomics insert) was plated to ensure that the concentration of antibiotic used entirely inhibited the growth of clones with only pZE21. The surviving colonies from each antibiotic selection were collected by adding 1500 μl of LB-Kan with 15% glycerol and then gently scraped the colonies with an L-shaped spreader from the agar plate. The slurry of antibiotic-resistant clones was removed from the surface of the plate and then stored at −80 °C before sequencing them with the Illumina NextSeq platform.

#### Sequencing, assembly, and annotation of antibiotic resistance genes

The plasmid DNA-containing antibiotic-resistant mgDNA fragments were extracted from functionally selected clones using the QIAprep Spin Miniprep Kit (Qiagen) and prepared for sequencing with a Nextera protocol as described above. The samples were submitted for sequencing using an Illumina NextSeq platform (2×150 bp reads). Reads from each antibiotic selection were assembled into contigs using PARFuMS [37], a tool specifically designed for high-throughput assembly of resistant-conferring DNA fragments from functional selections. Of note, selections were excluded from analysis if (i) the number of contigs assembled was 10 times more than the total number of colonies or (ii) more than 200 contigs were assembled. Contigs were also filtered based on length (> 500 bp).

The total number of contigs obtained was 7020, and in total, 16,334 open reading frames (ORFs) were predicted in these contigs using the gene finding algorithm Prodigal [45]. These ORFs were annotated following a hierarchical approach, where the ORFs were first searched against BLAST-based ARG databases (CARD [46], ResFinder [46], and AMRFinder-Prot [47]) with high percent identity (>95%) and coverage (>95%), and then the remaining ORFs were annotated using HMM-based ARG databases (Resfams [48], AMRFinder-fam [47]). This AMR gene annotator (*resAnnotator.py*) pipeline for the sequential annotation of ARGs using BLAST and HMM approach is available on GitHub. Overall, 1233 complete sequences were assigned using the *resAnnotator.py* pipeline. Percentage identity of 1233 ARGs was examined via a BlastP query against both the NCBI protein Non-Redundant (NR) database (retrieved 10 January 2020) and a combined database of all ARG proteins from CARD, NDARO to identify the top local alignment. The best hit identified using BlastP was then used for a global alignment using the needle program with the following non-default parameters: *-gapopen-10 -gapextend=0.5*. Twenty-two AMR genes did not have any homologs in known AMR sequence databases.

### Quantification of antibiotic resistance genes in metagenomes

AMR gene relative abundance was quantified using ShortBRED [49] v0.9.4. A ShortBRED marker database was built from 7921 antibiotic resistance proteins that were used as a protein of interest for the identification of marker families using *shortbred_identify.py*. Default parameters were used with the exception for *-clustid* 0.95 (see Additional file 1: Supplementary Note A for more information on 95% sequence identity clustering). Uniref90 [50] was the reference masking protein database (Additional file 1: Fig. S1). These protein sequences include ARG sequences from the Comprehensive Antibiotic Resistance Database (CARD) [46], the NCBI-AMR database [47], and antibiotic resistance proteins identified using functional metagenomics in this cohort as well as from previous studies [37–44, 51]. This resulted in a database consisting of 6585 unique marker sequences representing 2331 AMR gene families. These AMR gene families were then manually curated, and the entries with the following criteria were removed from analysis consideration because they would not be confidently expected to provide resistance based solely on a short-read marker (e.g., when that gene would require other components to provide phenotypic resistance, or when short-read markers would not distinguish between susceptible vs resistant versions of an antibiotic target):

1. Genes associated with global gene regulators, two-component system proteins, and signaling mediators (e.g., blaZ, vanS-vanR, mecI, mepR, gadW, marR)
2. Genes encoding subunits that are part of multiple efflux pumps (e.g., tolC, oprM, opmD)
3. Resistance via mutation in genes (e.g., resistance to antifolate drugs via mutations in dhfr, resistance to rifamycin via mutation in rpoB)
4. Genes conferring resistance by modifying cell wall charge (e.g., mprF)
5. Genes that reduce permeability (omp38, tmrB) or confer resistance through overexpression (e.g., thymidylate synthase)
6. General efflux pumps that came through functional selections (MFS-type, ABC-type)

The relative abundance of AMR gene families was quantified by mapping reads to the filtered set of marker sequences using *shortbred_quantify.py*. ShortBRED hits were filtered out if they had counts lower than 2 or mean reads per kilobase million (RPKM) lower than 0.001. The filtered list of markers is given in Additional file 2: Table S8.

### Metagenome profiling and assembly

Microbial taxa relative abundance was calculated using MetaPhlAn2 [52] (repository tag 2.6.0). Contig assembly was done using the de novo assembler SPAdes v3.14.0 [53]. Assemblies were annotated using our in-house AMR gene annotator called resAnnotator.py which includes CARD [46], Resfinder [54], NCBI-AMR [47], and Resfams [48]. Assemblies were also annotated with Prokka [55]. The BLAST+ command line tool (blastn) [56] was used to compare the *mcr-1* plasmid to our contig containing *mcr-1*. FastANI [57] v1.3 was used for average nucleotide identity comparisons between assembly GCA_004135815.1 (a CRE resistant *E. coli* isolated from stool from a patient with gastroenteritis in 2014 at Maharaj nakorn Chiang Mai hospital) and our draft genome assembly and for comparisons between our assembled *mcr-1* containing plasmid and NCBI Reference Sequence NZ_CP034405.1 (a plasmid sequence from the CRE resistant *E. coli* isolate). The BioSample for this isolate is SAMN10531954.

### MGE element profiling

Annotations with the following keywords were pulled from the functional metagenomic assemblies: transposase, transposon, integrase, integron, conjugative, conjugal, recombinase, recombination, mobilization, and phage. These elements were counted as putative mobile genetic elements. The same keywords were used in the analysis of putative mobile genetic elements from whole metagenome assemblies.

### Comparisons to other shotgun metagenomic data

The cohort of 110 Indian residents we compared to was published by Dhakan et al. in 2019 [58]. Fecal samples from this cohort were frozen within 30 min of collection and were subjected to whole metagenome shotgun sequencing. This cohort was selected because it includes a wide age range (average age of 29.72 with a standard deviation of 17.41) and samples from North-Central and South India, providing a more complete picture of the resistome than studies focusing only a single area or age range. Additionally, all travelers to South Asia in our study visited India, making it the most appropriate comparison country for this study. The data from Dhakan et al. can be accessed from BioProject PRJNA397112 or from [10.5524/100548].

### Statistical analysis and data visualization

**Fig. 1:**
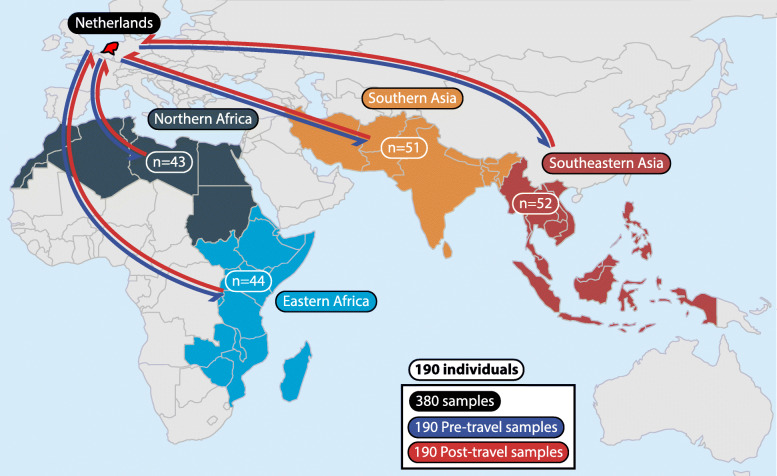
Destinations for Dutch travelers. A total of 190 Dutch individuals’ gut microbiomes were sampled before and after traveling (380 total samples) to 4 different subregions (Northern Africa, Eastern Africa, Southern Asia, and Southeastern Asia)

Statistical analysis was conducted in R [59] version 3.6.2. Visualizations were made using ggplot2 [60] version 3.1.0, ggpubr [61] version 0.2.5, and cowplot [62] version 1.0.0. Figures 1 and 9b were made using sf [63] version 0.1.8 and spData [64] version 0.3.3 with post-processing in Adobe Illustrator [65] version CC 2020 (24.0.2). Sankey networks were generated using networkD3 [66] version 0.4 with the *sankeyNetwork* function. Alignment visualization for *mcr-1* in Fig. 9c was made using genoPlotR [67]. Dirichlet multinomial mixture models [68] were made using DirichletMultinomial [69] version 1.26.0. For each clustering model (all samples together, only T0 samples, and only T1 samples), we did 50 iterations of clustering with different starting seeds. For each of these 50 iterations, we started with 1 cluster and stopped at a maximum of 25 clusters. Laplace approximations were generated for each cluster model, and the cluster model with the most evidence by this metric was chosen for further analysis (see source data of Figs. 3b and 5b for AIC and Laplace approximations for all clustering models). Samples were grouped with their best-matched cluster (see source data of Figs. 3b and 5b for cluster matching probability for each sample). Linear mixed-effects models were implemented with lme4 [70] version 1.1-21 (*lmer* function). Models were assessed using report [71] version 0.1.0 and performance [72] version 0.4.4. Vegan: Community Ecology Package [73] version 2.5.6 was used for the canonical analysis of principal coordinates [74] (*capscale* function), α- and β-diversity calculations (*diversity* and *vegdist* functions), and PERMANOVA tests (*anova.cca* function). Dabestr [75] version 0.2.3 was used for bootstrapping samples and calculating confidence intervals from bootstrapped samples. Linear models were implemented with lme4 [70] version 1.1.21 (*lmer* function). MaAsLin2 was used for modeling resistome data with metadata and taxonomic variables [76]. Subject_ID was used as a random effect for all models, and travel destination was also included as a random effect for the model that included all other metadata variables. Confidence intervals for non-bootstrapped samples were calculated using Rmisc [77] version 1.5 (*group.CI* function). Multinomial tests were calculated using the *multinomial.test* function from EMT [78] version 1.1. Stats (base R) version 3.6.2 was used for statistical calculations. The *wilcox.test* function was applied with *paired=T/F* as appropriate. The *fisher.test* function was for Fisher’s exact comparisons. The *binom.test* function was for binomial tests. The *p.adjust* function was applied where appropriate to correct for multiple hypothesis testing with *method=“fdr”* (Benjamini-Hochberg [79]). *p* values lower than machine precision of 2.220446e−16 are reported as *p*<2e−16. The *aov* function was used for the analysis of variance, and the *TukeyHSD* function was used for the analysis of variance significance testing. The *sqrt* function was used for square root transformations. Log transformation was implemented using a custom log function.

LOG <- function(*x*) {*y* <- replace(*x*, *x* == 0, min(*x*[*x*>0])/2); return(log10(*y*))}

## Results

### Cohort description and generation of a functional metagenomics augmented resistance marker database

To understand the effects of travel on the human gut resistome, we conducted whole metagenome shotgun sequencing and analysis on fecal samples from 190 Dutch individuals collected immediately before and after they traveled internationally to 4 different geographic regions (Fig. 1 and Additional file 2: Table S1). Our cohort visited Northern Africa (*n*=43), Eastern Africa (*n*=44), Southern Asia (*n*=51), and Southeastern Asia (*n*=52), yielding 380 samples (190 before travel and 190 after travel). A total of 174 study participants denied using antibiotics during the observation period while 10 participants claimed antibiotic use (6 participants answered unknown). The majority (*n*=170) were traveling on holiday, with a minority traveling for business (*n*=6), to visit relatives (*n*=4), and for religious purposes (*n*=10). Participants were adults with a median age of 50.7 (IQR 32.5–59.2) years.

To improve on AMR gene detection offered by conventional AMR databases, we used functional metagenomics. Functional metagenomics is a culture-independent method for identifying AMR genes from a metagenomic sample which, when expressed in a heterologous host, would enable this previously susceptible host to gain phenotypic resistance to an antibiotic [30, 37–44, 80]. In our protocol, we shotgun-cloned metagenomic DNA into an expression vector and transformed the resultant metagenomic expression libraries into *E. coli* indicator hosts. These *E. coli* transformant libraries were then screened against antibiotics at selective concentrations, and the cloned insert DNA in surviving transformants was sequenced to identify open reading frames that confer phenotypic resistance to the normally susceptible host. Here, we refer to AMR genes identified by this method as “functionally discovered AMR genes.”

We pooled our cohort stool samples within travel destinations to make 21 functional metagenomics libraries, which we screened against 15 antibiotics (Additional file 1: Fig. S1 and Additional file 1: Supplementary Note A) [30, 37]. These libraries yielded resistant transformants for every antibiotic screened except meropenem. By combining sequences from known AMR gene databases (CARD [46], NCBI-AMRFinder [47]) and from our functionally discovered AMR genes, we generated a custom ShortBRED [49] database with 6585 marker sequences corresponding to 2331 AMR gene families.

### Travel increases AMR gene abundance and α-diversity but decreases β-diversity

**Fig. 2:**
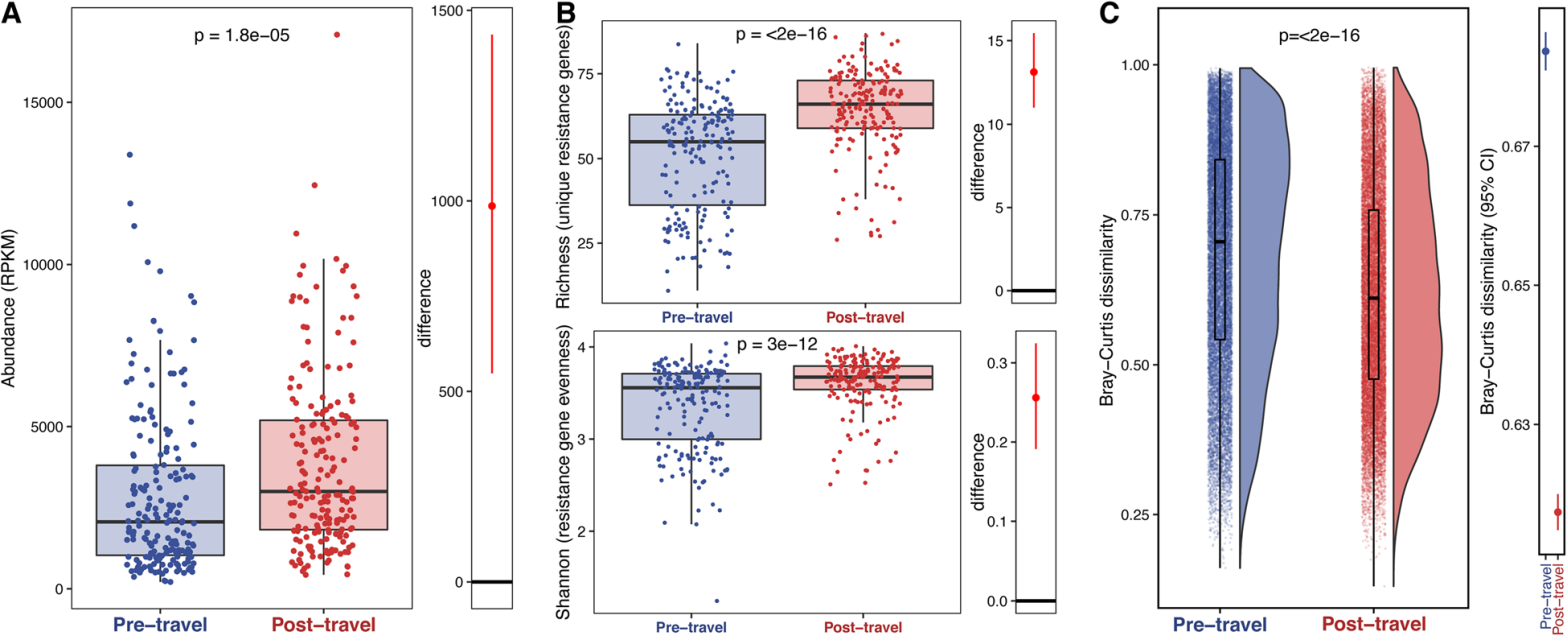
AMR gene abundance and α-diversity increases with travel and AMR gene β-diversity decreases. **a** The left panel shows the AMR gene abundance in RPKM. Each point is a sample, and the boxes are the medians with interquartile ranges for the pre-travel samples in blue and the post-travel samples in red. The *p* value (paired-sample *t* test) for the comparison is given at the top of the panel. The right panel shows the difference between the bootstrapped distributions of the post- and pre-travel samples. The red line gives the 95% confidence interval for the difference, and the point gives the estimate. **b** AMR gene α-diversity is measured by richness (top left panel), and Shannon Index (bottom left panel) is compared between the pre-travel (blue) and post-travel (red) samples. Each point corresponds to a given sample, and each box gives the median and interquartile range for the distribution. The *p* value (paired-sample *t* test) for the comparison is given at the top of each panel. The panels to the right of the boxplots show the difference between the bootstrapped distributions of the post- and pre-travel samples. The red line gives the 95% confidence interval for the difference and the point gives the estimate. **c** AMR gene β-diversity measured by Bray-Curtis dissimilarity is compared between the pre-travel (blue) and post-travel (red) samples. Each point is a comparison between two samples within the same time point group. The distributions are shown to the right of the points, and boxplots showing the median and interquartile ranges are overlaid on top of the points. The *p* value by paired Wilcoxon test for the comparison is shown near the top. In the right panel, the lines show the 95% confidence intervals, and points show the mean values for the pre- (blue) and post-travel (red) Bray-Curtis dissimilarity distributions. Source data is provided in the source data file (Additional file 3)

We used our custom ShortBRED database to profile the gut resistome in our 380 Dutch traveler samples. We then compared the pre- and post-travel samples for AMR gene abundance and diversity. AMR gene abundance in the gut microbiome was significantly higher (*p*=1.8e−5 [paired sample *t* test]) in the post-travel compared to the pre-travel samples (Fig. 2a), indicating that travel may enrich the microbiome for AMR determinants. This increase in abundance was matched by increased α-diversity (Fig. 2b) measured by unique AMR genes (richness, *p*<2e−16 [paired sample *t* test]) and by the evenness of AMR genes in the resistome (Shannon index, *p*<3e−12 [paired sample *t* test]). These results suggest that travelers are acquiring new AMR genes abroad.

Linear mixed-effects modeling of AMR gene abundance and α-diversity measured as richness (unique genes) showed that while the two measurements are significantly related (*p*<0.001), pre- or post-travel state significantly impacts AMR α-diversity (*p*<0.001) even when AMR gene abundance is accounted for (Additional file 2: Tables S2-S4 and Additional file 1: Supplementary Note B). Time point also had a larger effect on α-diversity (measured as richness) than it did on resistance gene abundance. These results are consistent with international travel as a driver of new AMR gene acquisition.

While AMR gene α-diversity increased following travel, resistome β-diversity (Bray-Curtis dissimilarity) between samples decreased (*p*<2e−16 [paired Wilcoxon test]) (Fig. 2c). These results suggest that the pressure of travel on the resistome may increase resistome similarity between individuals despite their different destinations. This finding could result from the acquisition of similar AMR genes.

### Unsupervised clustering separated pre- and post-travel samples into distinct subclusters, suggesting composition differences

**Fig. 3:**
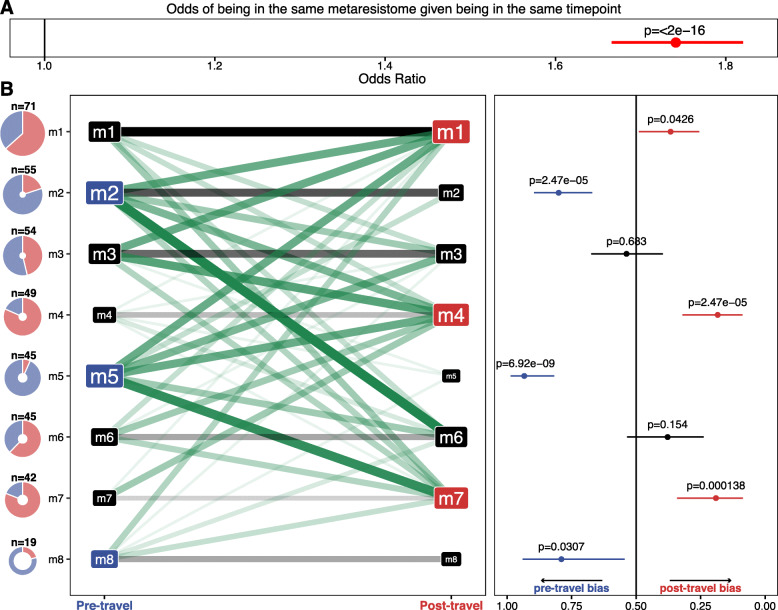
Travel outweighs subject effects in shaping resistome architecture. **a** 95% confidence interval (red line segment), odds ratio (red point), and *p* value calculated by Fisher’s exact test for samples with the same time point being drawn from the same metaresistome. The black vertical line at 1 shows the expected result under the null. **b** Each row in this plot corresponds to a metaresistome (m1–m8) in a Dirichlet multinomial mixture model of all traveler samples. The pie charts on the left are proportional in size to the number of samples in each metaresistome. The fill of the chart corresponds to the number of individuals in the time point (pre-travel in blue and post-travel in red). The network shows the number of individuals that transition from any model to any other model following their return from abroad. The black lines indicate staying within the same model, and the green lines indicate transition from one model to another model. The thickness and opacity of the lines correspond to the number of people following that transition path. Node label sizes correspond to the number of individuals in the model from the time point. Nodes filled in blue are significantly enriched in pre-travel samples, and nodes in red are significantly enriched in post-travel samples. The right panel shows the estimates (points) and 95% confidence intervals (lines) for binomial tests of bias for pre- or post-travel samples. *p* values for the comparison (FDR-corrected binomial test) are given above the lines. The expected estimate under the null model is given by the dark black line at 0.5. Source data for all panels is provided in the source data file (Additional file 3)

Dirichlet multinomial mixture models [68], an unsupervised method for clustering and modeling metagenomics data, revealed significant bias for samples from the same collection time point to group in the same metaresistome (*p*<2e−16 [Fisher’s exact test]) (Fig. 3a). Each metaresistome is a multinomial parameter probability vector, fit from a Dirichlet prior, over the resistance genes detected in our cohort. Together, the metaresistomes are the set of possible probability distributions that could result in our 380 samples using multinomial random draws. Thus, samples associated with the same metaresistome can be thought of as being drawn from the same underlying probability distribution.

Of the 8 metaresistomes in the best fit mixture model, 6 showed a significant bias to either the pre-travel (*n*=3) or post-travel (*n*=3) time point (Fig. 3b). Since each subject has two samples, we determined if an individual’s pre- and post-travel samples grouped in the same metaresistome. Instead, we observed 150 travelers (79%) switched metaresistomes, indicating a travel-specific effect in addition to subject random effects.

Since we have underlying AMR gene probability distributions for each metaresistome in our final mixture model, we can directly compare the models together. The post-travel-biased metaresistomes were characterized by higher α-diversity and lower β-diversity (Additional file 1: Fig. S2), mirroring the results we observed for the samples considered individually.

Prior studies of non-travel resistome perturbations [42, 81, 82] have used supervised clustering from Bray-Curtis dissimilarity [74, 83] to determine the group significance to resistome composition. Supervised clustering of our Dutch traveler resistomes also revealed significant separation (*p*=2e−4 [permanova]) between the pre-travel and post-travel samples (Additional file 1: Fig. S3A). However, the 8 optimal metaresistomes from the Dirichlet multinomial mixtures and the differences in the AMR gene diversity between metaresistomes suggest subclusters exist within the pre-travel and post-travel time points.

### Destination-specific resistome signatures explain metaresistome subclustering

**Fig. 4:**
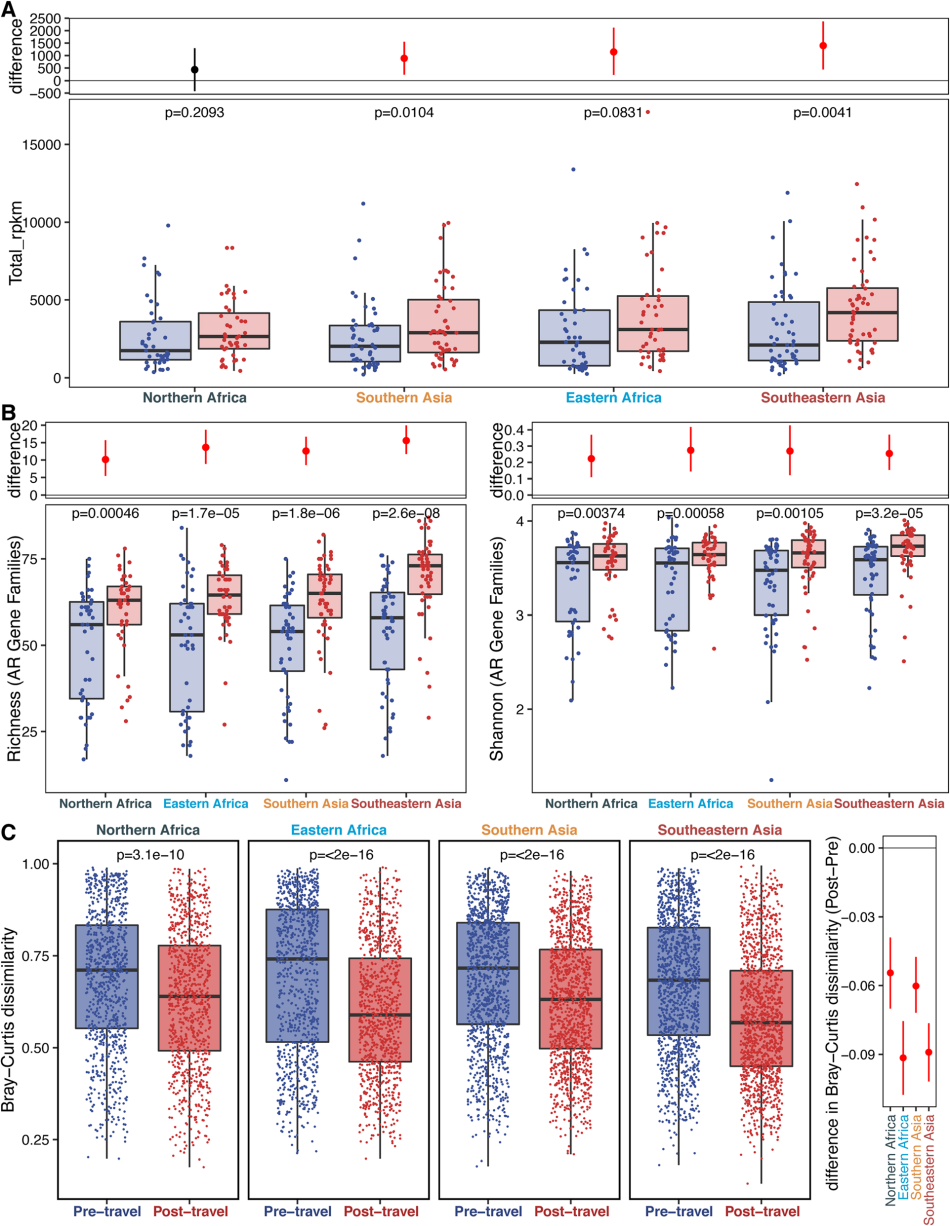
Travelers to different destinations cluster separately by resistome composition but show similar trends by abundance and diversity metrics. **a** The bottom panel shows the comparisons of AMR gene abundance before and after travel to the four subregions in this study. Points correspond to samples, and boxes give the median and interquartile ranges. Pre-travel is shown in blue, and post-travel is shown in red. The *p* values (FDR-corrected paired Wilcoxon tests) for comparisons within the region between the pre- and post-travel samples are shown above each comparison. The top panel shows the difference between the bootstrapped distributions of the post- and pre-travel samples. The red line gives the 95% confidence interval for the difference, and the point gives the estimate. **b** AMR gene α-diversity is measured by richness (left), and Shannon Index (right) is compared by region between the pre-travel (blue) and post-travel (red) samples. Each point corresponds to a given sample, and each box gives the median and interquartile range for the distribution. The *p* values (FDR-corrected paired Wilcoxon test) are above each comparison. The panels above show the difference between the bootstrapped distributions of the post- and pre-travel samples for each destination. The red line gives the 95% confidence interval for the difference, and the point gives the estimate. **c** The left panel compares the β-diversity for pre-travel (blue) and post-travel (red) collections for the four travel destinations. The points are pairwise Bray-Curtis dissimilarity between two samples, and the boxes represent the median and interquartile ranges of the distributions. *p* values (paired Wilcoxon test) are given above each comparison. The right panel shows the difference between the bootstrapped dissimilarities of the pre- and post-travel groups. The lines give the 95% confidence interval for the difference, and the point gives the estimate. Source data for all panels is provided in the source data file (Additional file 3)

Though all four destinations had increased AMR gene abundance (Fig. 4a) and α-diversity (Fig. 4b), the magnitude of these differences varied and broadly agree with clinical isolate resistance data published by the Center for Disease Dynamics, Economics, and Policy (Additional file 2: Table S5 and Additional file 1: Supplementary Note C). Resistome α-diversity was significantly higher for individuals returning from Southeastern Asia than from the other three destinations (Additional file 1: Fig. S4). Individuals traveling to the same subregion also had decreased interindividual resistome β-diversity (*p*=0.016 [unpaired Wilcoxon test]), suggesting that having the same travel destination makes traveler resistomes more similar (Additional file 1: Fig. S5 and Additional file 1: Supplementary Note D). These β-diversity decreases were significantly larger in travelers returning from Southeastern Asia and Eastern Africa than Northern Africa and Southern Asia (Fig. 4c). Thus, individuals returning from Southeastern Asia and Eastern Africa had more similar AMR profiles to other travelers to the same destination than individuals returning from Northern Africa and Southern Asia.

**Fig. 5:**
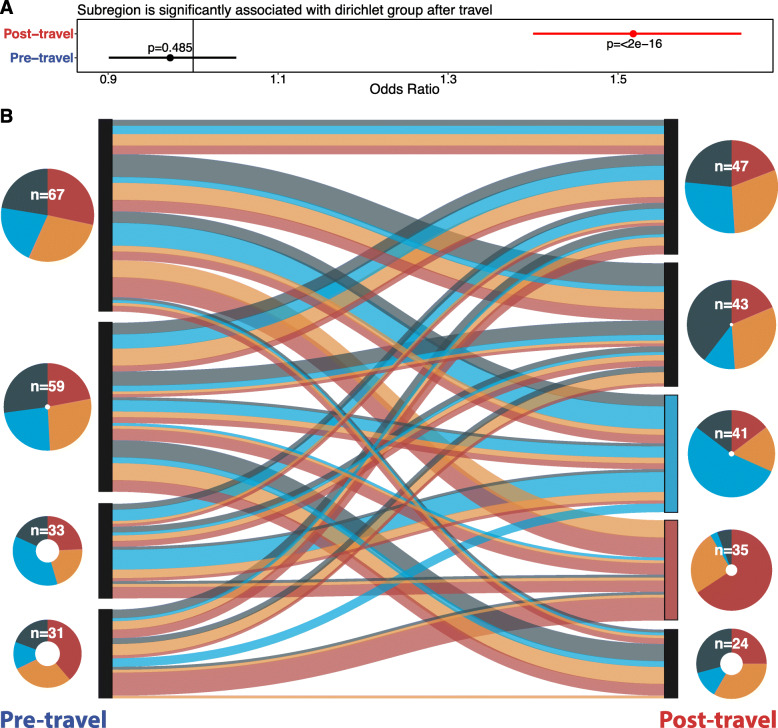
Travelers’ resistomes group significantly by region after travel, and Southeastern Asia and Eastern Africa have the strongest signature. **a** 95% confidence intervals, odds ratios, and *p* values for the samples with the same destination being drawn from the same metaresistome. Fisher’s exact tests were done for this comparison within the time point (*y*-axis). The black vertical line at 1 shows the expected result under the null. Source data for all panels is provided in the source data file (Additional file 3). **b** The left of this Sankey diagram has models built from the pre-travel samples, and the right has models built from the post-travel samples. Each model has a pie chart that shows the number of samples in the model (total of 190 for each time point), and these pies are divided by destination. The lines connecting the pre- and post-travel models are colored according to region (dark blue is Northern Africa, light blue is Eastern Africa, orange is Southern Asia, and red is Southeastern Asia), and their thickness is proportional to the number of samples that follow that path

To interrogate these region-specific effects, we rebuilt Dirichlet multinomial mixture models after separating the pre-travel and post-travel samples. Separating the time points removes possible random effects due to subject identity allowing a narrower focus on destination. This analysis yielded 9 metaresistomes (4 in the pre-travel samples and 5 in the post-travel samples). Though the pre-travel metaresistomes did not show significant bias by destination (*p*=0.485 [Fisher’s exact test]), the post-travel metaresistomes had a strong regional bias (*p*<2e−16 [Fisher’s exact test]) (Fig. 5a). These results also appeared in supervised clustering where destination significantly distinguished samples (Additional file 1: Fig. S3B-C) after travel (*p*=4e−4 [permanova]) but not before travel (*p*=0.7021 [permanova]). This demonstrates that individuals traveling to the same destination are far more likely to have their post-travel samples cluster in the same metaresistome than their pre-travel samples.

Considering these destination signatures, we wanted to determine if the travelers’ resistomes looked similar to resident gut resistomes in their travel destinations. We used shotgun metagenomic reads from a recently published cohort of fecal microbiomes from the Indian subcontinent [58]. After profiling the Indian resistomes using our ShortBRED AMR protein marker database, we found that the Dutch subjects returning from Southern Asia (which includes India) had resistomes that were more similar to the Indian resistomes compared to subjects returning from the other three subregions (Northern Africa *p*=2.2e−10; Eastern Africa *p*<2e−16; Southeastern Asia *p*<2e−16 [unpaired Wilcoxon test]) (Additional file 1: Fig. S6 and Additional file 1: Supplementary Note E).

The grouping effect of destination was strongest for Eastern Africa and Southeastern Asia (Fig. 5b). This finding matches the previous results (Fig. 4c) where interindividual resistome β-diversity was lower in subjects returning from these two destinations. We can see from these analyses that the destination-specific effects result in individuals returning from the same destination having similar post-travel resistome states despite diverse pre-travel states.

### AMR gene abundance increases and acquisitions during travel are concentrated in several AMR gene families and resistance mechanisms

We found a positive correlation between prevalence and abundance (*p*<2e−16) for AMR genes in our Dutch traveler cohort (Additional file 1: Fig. S7), but this correlation varies by AMR mechanism. Efflux (*p*=6.73e−5), inactivation (*p*=2.76e−4), and target protection (*p*=7.87e−8) all had significant positive correlations, and the trend for antibiotic target alteration was also positive. In contrast, the trend for antibiotic target replacement is nearly flat, showing that target replacement genes we detected in gut resistomes do not have a corresponding increase in abundance when they are prevalent in more samples.

**Fig. 6:**
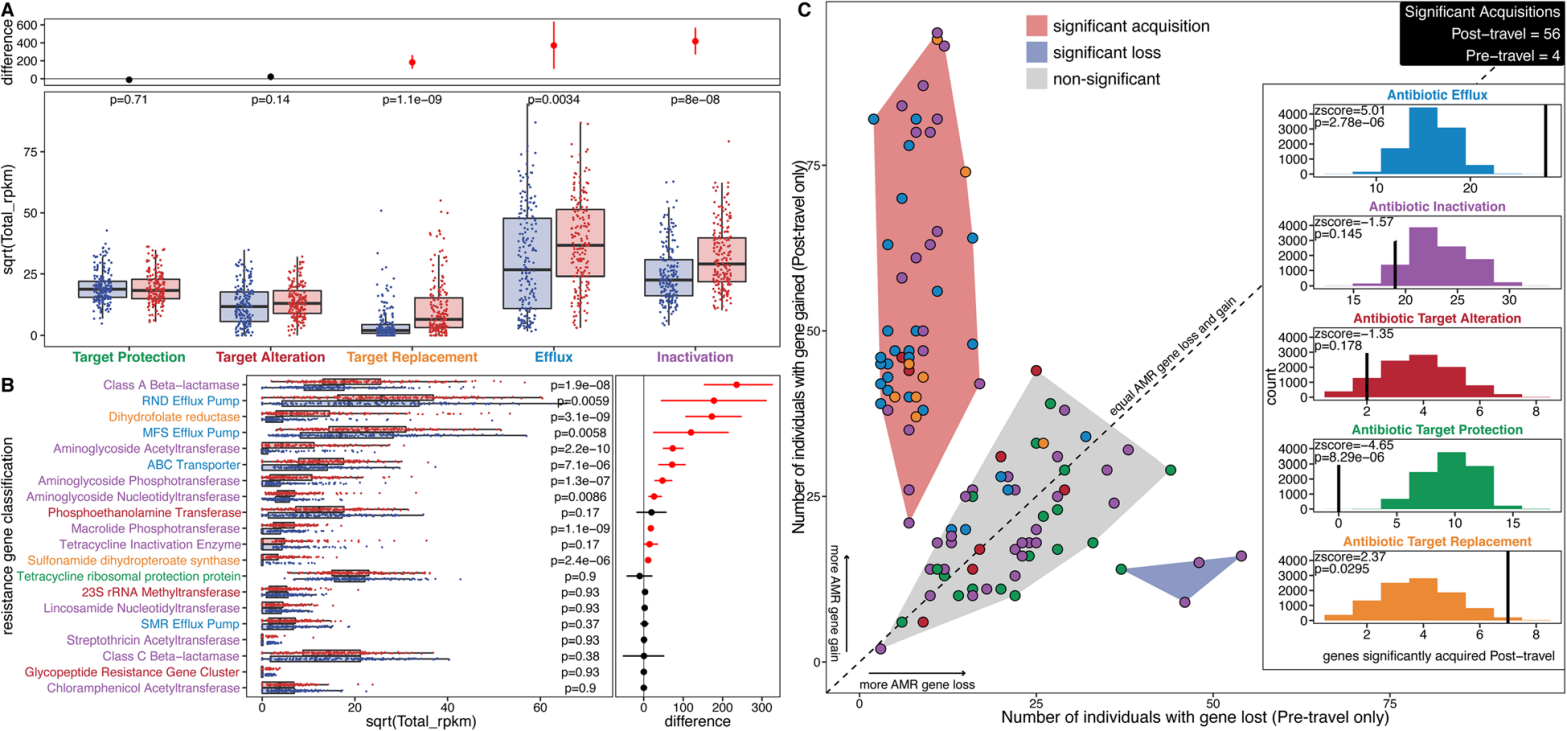
AMR gene abundance changes and acquisitions are unequal across AMR mechanisms. **a** AMR mechanism abundance is compared between pre-travel (blue) and post-travel (red) samples. Each point is a sample, and the boxes represent the median and interquartile range. *p* values (FDR-corrected paired Wilcoxon test) for the comparisons are given near the top of the panel. The top panel shows the difference between the bootstrapped distributions of the post- and pre-travel samples. The lines give the 95% confidence interval for the difference, and the point gives the estimate. AMR classes where the 95% confidence interval does not cross 0 are red. **b** AMR class abundance is compared between pre-travel (blue) and post-travel (red) samples. Each point is a sample, and the boxes represent the median and interquartile range. *p* values (FDR-corrected paired Wilcoxon test) for the comparisons are given near the top of the panel. The top panel shows the difference between the bootstrapped distributions of the post- and pre-travel samples. The lines are the 95% confidence interval for the difference, and the point is the estimate. AMR classes where the 95% confidence interval does not cross 0 are red. **c** AMR gene acquisitions or losses after travel. Each point is an AMR gene, and points are filled in according to their AMR mechanism. The *x*-axis is the number of individuals that had the gene in the pre-travel time point, but not in the post-travel time point. The *y*-axis is the number of individuals that had the gene in the post-travel time point, but not in the pre-travel time point. The red-shaded region spans significantly acquired AMR genes, the blue-shaded region spans significantly lost AMR genes, and the gray-shaded region spans genes that were not significantly acquired or lost. The diagonal line is the null of equal losses and gains for an AMR gene. The inset panel shows which AMR mechanisms were significantly acquired during travel by permutation testing. The colored histograms show the expected distribution according to 10,000 permutations, and the black vertical lines show the observed value (points in the red-shaded region of the main plot). The *z*-score and the FDR-corrected *p* value for the comparison of observations to their expected distribution are given in the top left of each plot. Source data for all panels is provided in the source data file (Additional file 3)

We next assessed if the abundance of these mechanisms changed following travel (Fig. 6a). The abundance of genes encoding for target replacement (*p*=1.1e−9), efflux (*p*=3.4e−3), and inactivation (*p*=8.0e−8) of antibiotics all significantly increased after travel. This indicates that at the level of AMR mechanisms, there is a significant effect of travel in structuring the gut resistome. By further classifying the AMR genes families into gene classes defined by CARD ontology, we observed that 11 of 20 detected classes had increased abundance in the post-travel samples compared to the pre-travel samples (Fig. 6b). These data demonstrate that travel-related resistance gene increases are not limited to those identified by culture-based analysis. The strongest effect was seen in class A β-lactamases which inactivate several clinically important antibiotics, though we did not observe class A carbapenemases. This is consistent with the lack of resistant transformants observed against meropenem in our functional metagenomic libraries.

We detected 56 AMR genes with significant evidence of acquisition after travel, compared to only 4 showing significant loss following travel (Fig. 6c and Additional file 1: Fig. S8), highlighting the heavy bias of AMR gene acquisition in the post-travel samples. AMR genes for antibiotic efflux (*p*=2.78e−6 [permutation test]) and for antibiotic target replacement (*p*=0.0295 [permutation test]) were both highly enriched in the significantly acquired set of genes. In contrast, AMR genes for antibiotic target protection (*p*=8.29e−6 [permutation test]) were completely absent in the significantly acquired genes (far less than predicted under a null model).

The diversity of AMR genes with significantly increased abundance and acquisition post-travel demonstrates the importance of expanding AMR analysis beyond ESBLs to the entire gut resistome. For example, we detected two variants of *tetX*, an antibiotic-inactivating monooxygenase which confers resistance against all clinically relevant tetracyclines, including last-resort antibiotics like tigecycline, eravacycline, and omadacycline [84, 85]. Tetracycline inactivation AMR genes increased in abundance after travel (Fig. 6b), but the acquisition was only significant for one of two *tetX* variants (Additional file 1: Fig. S8). The variant of *tetX* encoded in NCBI-AMR was not significantly acquired during travel (0.59 CI [0.406–0.763], *p*=0.523 [binomial test]), while the variant of *tetX* discovered in our functional selections was significantly acquired during travel (0.75 CI [0.551–0.893], *p*=0.0247 [binomial test]).

AMR gene acquisitions were also significant when accounting for gene abundance (Additional file 1: Supplementary Note F). Models with taxonomic covariates (Additional file 1: Fig. S9) built at both the broad AMR gene classification level (Additional file 1: Fig. S10-S12) and at the detailed single-gene level (Additional file 2: Table S6) all showed more AMR determinants associated with the post-travel time point. A model with all metadata included identified time point as a significant predictor of AMR gene abundance for 65 of the 121 AMR genes (Additional file 2: Table S7 and Additional file 1: Supplementary Note F). Travel duration had a weak but significant effect on AMR gene acquisition when all AMR gene acquisitions were considered together (Additional file 1: Fig. S13). When the 121 AMR genes were considered individually, increased travel duration only significantly correlated with *catA*, a chloramphenicol acetyltransferase (Additional file 2: Table S7).

### Travelers to Southeast Asia had the most AMR gene acquisition and Southeast Asian functional selections had high mobile genetic element burden

**Fig. 7:**
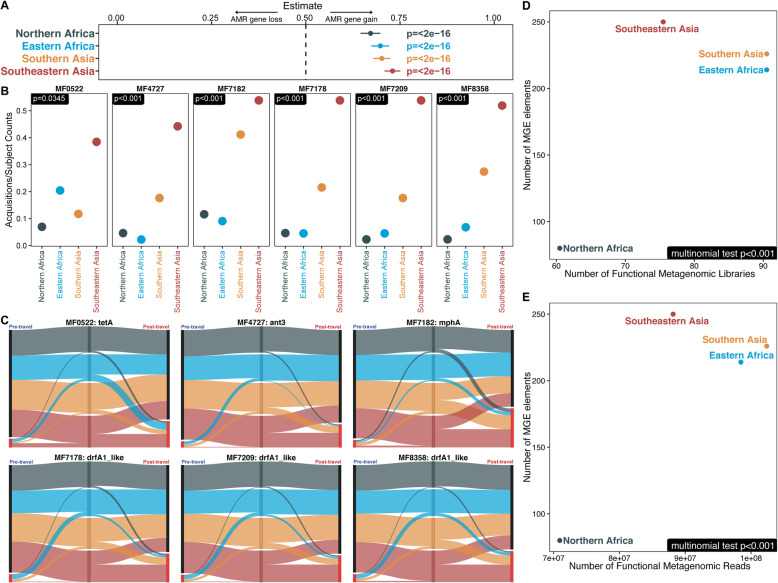
AMR gene acquisitions and mobile genetic elements differed by travel destination. **a** Significance of AMR gene acquisitions by travel destination. The lines show the 95% confidence intervals, and the points show the estimates of binomial tests for bias. Binomial tests were conducted by region for the number of acquired AMR genes and the number of lost AMR genes. Both acquisitions and losses were normalized by the number of individuals traveling to the region. *p* values (FDR-corrected) from this test are shown just below the dotted line at 0.5 indicating the null. Numbers lower than 0.5 indicate AMR gene loss, and numbers greater than 0.5 indicate AMR gene gain. **b** Genes that showed significant region-specific bias following multinomial testing. Points indicate their number of acquisitions normalized by the number of travel subjects, and *p* values are given in the top left. **c** Sankey diagram of AMR gene acquisitions by travel region. Black nodes are when the gene was not found, and bright red nodes indicate the gene was present. The width of all lines is proportional to the number of individuals following that path. **d**, **e** The number of MGE elements detected from the functional metagenomic libraries is plotted on the *y*-axis, and the number of input **d** libraries and **e** reads is on the *x*-axis. *p* values calculated by the FDR-corrected multinomial test are in the bottom left of each panel. Most *p* values in **b**, **d**, and **e** hit underflow and have been set to *p*<0.001. Source data for all panels is provided in the source data file (Additional file 3)

Every destination showed significant AMR gene acquisition (Fig. 7a), with travelers to Southeastern Asia having the highest AMR gene acquisition (0.73 CI [0.71–0.75], *p*<2e−16) and those visiting Northern Africa having the lowest AMR gene acquisition (0.67 CI [0.65–0.70], *p*<2e−16). Six of the 56 significantly acquired AMR genes identified in Fig. 6c were significantly associated with travel destinations (Fig. 7b, c). Travelers to Southeastern Asia had the most acquisitions normalized by the number of subjects traveling to the region, for all six genes. Three of these AMR genes were *dfrA1* variants, which confer resistance against trimethoprim. Each *dfrA1* variant had a fold change increase between 3.62 and 3.92 in prevalence in post-travel samples. Increases we saw in trimethoprim-sulfamethoxazole resistance genes (Figs. 6b and 7c) parallel results from Blyth et al. 2016 where 42% of post-travel *E. coli* isolates had new resistance against trimethoprim-sulfamethoxazole [15].

There was a bias for aminoglycoside resistance gene *ant3* to be acquired in Southeastern Asia, and a bias for the macrolide resistance gene *mphA* to be acquired in Southeastern and Southern Asia. *tetA* was the only AMR gene of these six with more acquisition events from Eastern Africa than from Southern Asia though Southeastern Asia still had the highest acquisition rate.

Genomic context like colocalized mobile genetic elements impact AMR gene spread [11, 19, 20, 86]. To search for AMR gene context, we assembled contigs from our travelers’ metagenomic samples and searched for putative mobile genetic element annotations adjacent to AMR genes. In these AMR-containing contigs, we detected a higher burden of putative mobile genetic elements in post-travel samples than in pre-travel samples (*p*=1.4e−10 [paired Wilcoxon test]) (Additional file 1: Fig. S14A). This difference was significant across all regions (Additional file 1: Fig. S14B), but not between travel destinations (Additional file 1: Fig. S15).

Destination differences did appear when we counted the number of mobile genetic element-associated annotations on contigs with AMR genes from our functional metagenomics data. We split these counts based on sample destination for the inputs to the functional metagenomics selections, and we found a significant association between subregion and the number of mobile genetic element annotations. This was true when we normalized by the number of input reads (Fig. 7d) or by the number of input libraries (Fig. 7e). Travelers to Southeastern Asia had the most mobile genetic element-associated annotations despite having fewer input reads and fewer input libraries. Though travelers to Southeastern Asia had the highest number of mobile genetic elements adjacent to AMR genes, travelers to Southern Asia and Eastern Africa also had comparable numbers. Travelers to Northern Africa had far fewer AMR gene-associated mobile genetic element annotations than the other three regions. This is concordant with our findings showing that travelers to Northern Africa also had lower AMR gene abundance and acquisition than other destinations.

Our results suggest that the colocalization of mobile genetic elements with AMR genes correlates with destination-specific resistance gene acquisition and demonstrate the importance of functional metagenomics data in detecting these differences. This fact is highlighted for subregion by the contrast between the lack of association with travel destination for MGE annotation counts across all assemblies (not necessarily colocalized with AMR genes) as presented in Additional file 1: Fig. S15 and the strong association with travel destination in Fig. 7d, e showing annotations in the functional selections where the mobile genetic elements are adjacent to AMR genes. The number of mobile genetic elements adjacent to AMR genes may contribute to the increases in AMR gene burden post-travel.

### qPCR detected high-risk AMR genes acquired by Dutch travelers

Concurrent with our comprehensive metagenomic resistome analysis, we specifically targeted 16 clinically important AMR genes in our samples by qPCR. Of these 16, four genes (*tetM*, *tetQ*, *ermB*, and *mefAE*) were present in all of the samples, and two genes (*qnrA* and *mcr-2*) were not present in any samples. We conducted acquisition analysis and destination bias analysis for the remaining 10 genes.

**Fig. 8:**
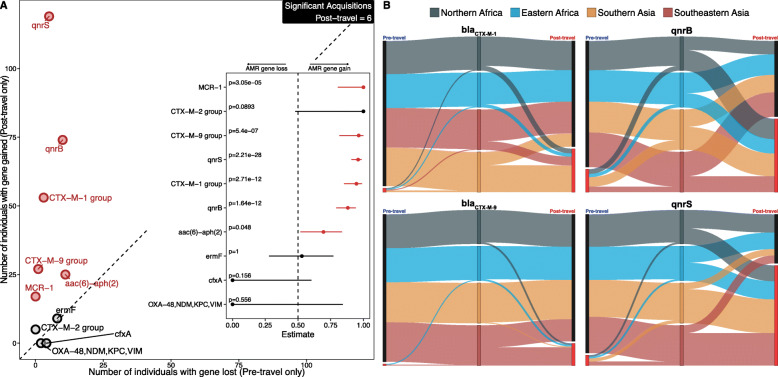
Quinolone resistance genes were acquired in regions with equal frequency, while β-lactam resistance genes had destination-specific acquisition. **a** AMR genes acquired or lost after travel detected by qPCR. Each point is an AMR gene. The *x*-axis is the number of individuals that had the gene in the pre-travel time point, but not in the post-travel time point. The *y*-axis is the number of individuals that had the gene in the post-travel time point, but not in the pre-travel time point. Significant acquired AMR genes are in red. The number of significant genes is tabulated in the top right. Non-significant genes are in black. The diagonal line is the null of equal losses and gains for an AMR gene. The inset panel gives the results from binomial tests of bias for AMR gene acquisition for the post-travel time point. The lines are 95% confidence intervals, and the points are estimates. *p* values (FDR-corrected binomial test) are given at the bottom of the plot for each gene. The dotted line is the expected value under the null. The lines and points are red if significantly acquired. **b** Sankey diagrams of significant gene acquisitions by travel region detected by qPCR. Black nodes are when the gene was not found, and bright red nodes indicate the gene was present. Ribbon colors correspond to the destination countries (dark blue is Northern Africa, light blue is Eastern Africa, orange is Southern Asia, and red is Southeastern Asia). The width of all lines is proportional to the number of individuals following that path. Source data for both panels is provided in the source data file (Additional file 3)

Acquisition analysis showed that 6 of the 10 AMR genes that were present in at least 1 sample were significantly associated with the post-travel time point (Fig. 8a). Notably, *mcr-1*, a plasmid borne colistin resistance gene, was found only in post-travel samples.

**Fig. 9:**
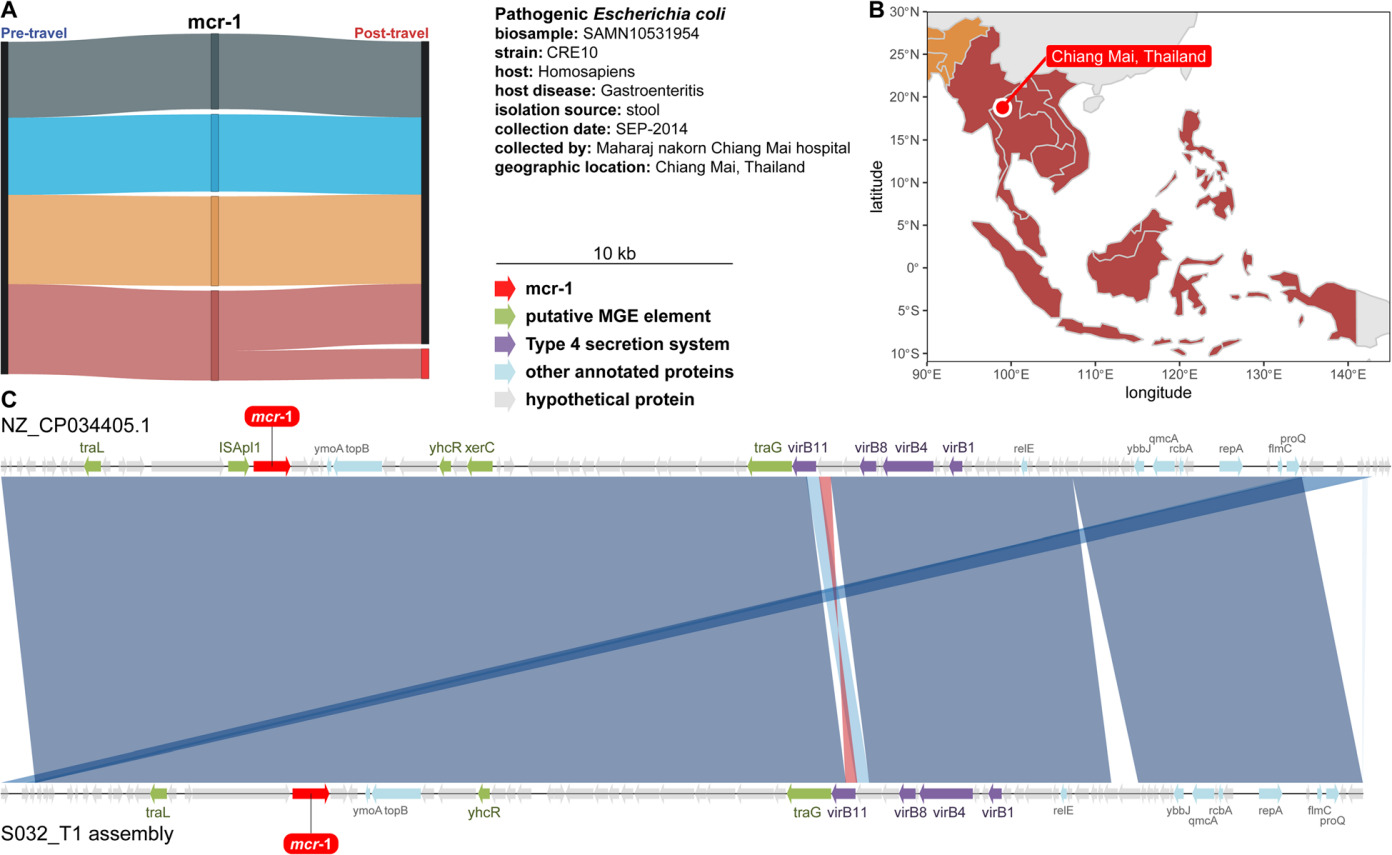
*mcr-1* containing contig from a Dutch traveler matched a plasmid sequenced from a gastroenteritis patient in the destination region. **a** Sankey network showing region-specific acquisition for *mcr-1*. **b** Map showing where the reference genome was isolated from a gastroenteritis patient. **c** Alignment between a plasmid from an *E. coli* isolated from a gastroenteritis patient in Chiang Mai and a contig assembled from a Dutch traveler’s gut microbiome. Source data for all panels is provided in the source data file (Additional file 3)

Quinolone resistance genes *qnrB* and *qnrS* were acquired in high proportion following travel to all four subregions (Fig. 8b), but *bla*_CTX-M-1_, *bla*_CTX-M-9_, and *mcr-1* had strong region-specific effects (Figs. 8b and 9a). Over 80% of *bla*_CTX-M-1_ and *bla*_CTX-M-9_ β-lactamase acquisitions were in travelers to Asia. *bla*_CTX-M-1_ was predominantly acquired in Southern Asia (61.8%), and *bla*_CTX-M-9_ was predominantly acquired in Southeastern Asia (82.1%). Uniquely, *mcr-1* was only acquired by travelers to Southeastern Asia (Fig. 9a).

### Dutch travelers to Southeastern Asia acquired *mcr-1*

We also observed that AMR genes in our cohort were often colocalized with mobile genetic elements. *mcr-1*, a plasmid-borne colistin resistance gene, was one of the most concerning genes we detected. Colistin is a drug of last resort for drug-resistant gram-negative pathogens used when other antimicrobial therapies fail [87, 88]. *mcr-1* is the first plasmid-borne resistance gene against colistin, and it is responsible for rapidly increasing colistin resistance observed over the last 4–5 years [20]. Eighteen of the 52 Dutch travelers to Southeastern Asia (34.6%) acquired *mcr-1* during our study.

To further investigate the *mcr-1* colistin resistance gene, we assembled contigs using the raw shotgun reads from the samples that were *mcr-1* positive by qPCR and annotated these contigs for *mcr-1*. One contig assembled from subject S032, a traveler returning from Vietnam, was positive (Fig. 9). Plasmidfinder 2.0 identified subject S032’s *mcr-1* containing contig as an IncI2 type plasmid (100% identity and 100% template length using the Enterobacteriaceae database) [89, 90]. A follow-up study [91] of the 6 isolates from Arcilla et al. [92] demonstrated that one ESBL-producing *E. coli* from a traveler returning from Vietnam also harbored *mcr-1* on an IncI2 type plasmid.

We searched NCBI for the best BLAST match of subject S032’s *mcr-1* containing contig, and the top hit (99.9% identity with 100% query coverage) was to a plasmid from gastroenteritis-causing *E. coli* (Fig. 9b) isolated in Chiang Mai, Thailand (data from BioSample SAMN10531954 and NCBI reference sequence NZ_CP034405.1). In both plasmids (Fig. 9c), *mcr-1* is flanked by a *tra* cluster of conjugon transfer genes, and *mcr-1* is also colocalized with a *virB* cluster type IV secretion system (T4SS). T4SS have known involvement in horizontal gene transfer [93–97]. There are prior reports of *mcr-1* colocalization with T4SS in plasmids [20, 98], though in those studies *mcr-1* was on different plasmid incompatibility types than IncI2.

## Discussion

Global AMR spread threatens decades of success in treating bacterial infections with antibiotics [6, 10, 99–101]. This problem is exacerbated by the worldwide spread of antibiotic-resistant bacteria and AMR genes by international travelers [8, 102]. Our investigation of 190 Dutch individuals’ gut resistomes before and after travel indicates international travel is a significant gut resistome perturbation and highlights the extent of AMR gene acquisition. We found the acquisition of previously unknown, functionally discovered AMR genes, increased AMR gene abundance, and increased resistome α-diversity in the post-travel samples. We also observed AMR gene colocalization with mobile genetic elements and identified travel destination-specific resistome signatures.

A study by Langelier et al. in 2019 reported on the resistome in 10 travelers to Asia or Africa [103]. Eight of these travelers went to Nepal, one went to Nigeria, and one went to Uganda. The authors sampled the subjects once before travel and thrice after travel; they found increased AMR genes against multiple antibiotic classes, including β-lactams, quinolones, and anti-folates. This increase in AMR genes after travel mirrors our results, and many of the AMR genes they identified were also detected in our study. Interestingly, in contrast to the results in Langelier et al., we saw increases in some tetracycline resistance genes and aminoglycoside resistance genes after travel. For the tetracycline resistance genes, this may be explained by our more detailed consideration of the resistance mechanism. We observed that while tetracycline inactivation mechanisms significantly increased in abundance after travel, tetracycline ribosomal protection mechanisms did not. In fact, none of the tetracycline ribosomal protection resistance genes was significantly acquired during travel. Our study-specific functional metagenomic selection database also provides higher sensitivity to detect AMR genes that may be underrepresented in conventional AMR databases. Indeed, 51 of the 121 (42.1%) AMR genes detected and compared in our analysis were from functional selections. The AMR genes identified in Langelier et al. are often found in commonly cultured clinical isolates and thus are well represented in conventional AMR databases.

An individual’s gut resistome response to travel perturbation may parallel the response from other non-travel gut perturbations, including hospitalization and antimicrobial treatment [104, 105]. In a 2017 study of healthy patients compared to antibiotic-treated patients hospitalized in an ICU in The Netherlands, Buelow et al. found that healthy patients had enriched *tetW* and *catA* [105]; both *tetW* and *catA* were also more likely to be found in our pre-travel than post-travel samples. In contrast, the antibiotic-treated ICU patient resistomes in the Buelow et al. study were enriched for AMR genes such as *erm* and an *aac*(6’) family gene, both of which were also acquired and increased in our post-travel samples. With antibiotic perturbation, the effects on the gut resistome can vary based on the spectrum of the antibiotic [41, 42, 81]. However, studies commonly observe an increase in resistome α-diversity and a decrease in β-diversity [81]. This is similar to our observations in response to travel perturbation. Additionally, some studies show a time dependence for AMR gene acquisitions and abundance increases [42, 81] paralleling the weak time dependence we show in our results. There are conflicting results if these antibiotic perturbations return to the initial state or leave persistent scars [42, 106]. Even if the travel-related resistome changes revert to baseline, it is possible that the AMR genes will be disseminated in the resident country before they are lost in the host.

The high-risk gene acquisitions we observed are concordant with qPCR-based research of endemic antibiotic resistance in our cohort’s travel destinations. In 2019, Bich et al. demonstrated *qnr* endemicity in Vietnam with 100% carriage of *qnr* by qPCR of fecal samples from 93 Vietnamese residents of the Ha Nam province [107]. This same study also found carriage of *bla*_CTX-M-1_ (38%) and *bla*_CTX-M-9_ (61%). These results correspond well with both the high acquisition rate we saw for these genes in individuals returning from Southeastern Asia and the *bla*_CTX-M-9_ predominance we saw in travelers returning from Southeastern Asia.

Our cohort’s mcr-1 Southeast Asian geographic acquisition bias is also consistent with Bich et al. where 88% of tested Vietnamese residents carried *mcr-1* [107]. In comparison, a culture-based study [92] by Arcilla et al. of ESBL-producing *E. coli* isolates from all 2001 participants (540 to Southeastern Asia) in the COMBAT study detected *mcr-1* in 6 *E. coli* isolates, indicating higher detection sensitivity for *mcr-1* using qPCR directly from the stool. These results are also comparable to another culture-based isolate study [27] where 20 of 412 returning US travelers yielded *mcr*-harboring *E. coli*.

In 2018, Wang et al. analyzed *mcr-1*-containing plasmids across a number of different bacterial isolates from around the globe [11]. China and Vietnam were the two countries with the most isolates harboring *mcr-1* plasmids, which corresponds to our detection of *mcr-1* in travelers to Southeastern Asia. The authors found that *mcr-1* initially mobilized to plasmids through an ISApl1 transposon. This is consistent with the reference plasmid in Fig. 9c.

Our *mcr-1* results advocate for a combined approach of AMR gene detection. Short-read shotgun metagenomic sequencing provided us with excellent data for understanding gut resistome composition changes, diversity changes, and AMR gene acquisitions due to travel, but only 1 of 18 (5.6%) *mcr-1* qPCR-positive stool samples we assembled yielded an *mcr-1* contig. However, we show that AMR gene contig assembly yields an important genomic context surrounding resistance genes that could have implications for understanding and modeling AMR gene spread. Contig assembly using short-read shotgun metagenomic sequencing may differ by AMR gene. For example, we successfully assembled *tetX* in 56 of 143 (39.2%) ShortBRED-positive samples. Future studies may employ chromosome conformation capture or long-read sequencing in concert with short-read sequencing to improve metagenomic assembly and give even more detailed genomic context to resistance gene detection directly from stool [28].

Our study design was optimized to understand the acquisition within travelers, and we do not have samples from travelers’ contacts while abroad. Pre-travel samples from our Dutch cohort also contain resistant bacteria and AMR genes, but our study is only equipped to show unidirectional gene transfer from the destination to the travelers; it is also possible that travelers could deposit AMR genes in their travel destinations. Future investigation into travelers’ contacts at home and abroad may resolve AMR gene transmission networks. We also observed that grouping samples by subregion better explains the sample composition than grouping by continent. It is possible that we are missing even more granular effects that would be found at the country or even city level [40, 108].

## Conclusions

We provide new data regarding the effect of international travel to low- and middle-income regions on the gut resistome of travelers from a high-income country. We show that such travelers acquire AMR genes abroad and carry these AMR genes back to their countries of origin. These AMR genes include both known clinically relevant AMR genes that are common in pathogens (e.g., bla_CTX-M_ and *mcr-1*) and functionally discovered AMR genes with no known homologs in the current databases. We also show AMR gene acquisition and carriage in the gut resistome is travel destination-specific with compositional signatures lasting at least until the traveler returns home. Interventions to reduce AMR burden in low- and middle-income countries with current high endemic AMR burdens may reduce traveler AMR gene acquisitions. Developments in risk stratification for AMR genes could help target such efforts [109].

## Supplementary Information

**Additional file 1:** Supplementary notes (A-F), supplementary table legends (S1-S9), and supplementary figures (S1-S15).

**Additional file 2:** Supplementary tables (S1-S9).

**Additional file 3:** Source data for all figure panels.

**Additional file 4:** Scripts that takes in community data matrix and outputs Dirichlet Multinomial model object.

**Additional file 5:** Takes in community data matrix and directory with Dirichlet Multinomial model objects and outputs data from the best fit model.

**Additional file 6:** Outputs a data table for AIC values of DMN models and makes aggregated AIC and Laplace plots.

## Data Availability

Source data for all figures is provided in the source data document (Additional file 3). Assembled functional metagenomic contigs and raw shotgun metagenomic reads have been deposited and released to NCBI SRA under BioProject ID PRJNA688274 [110]. Other publicly available data used in this project: Data from Dhakan et al. 2019 is deposited under BioProject PRJNA397112 [111]. Data from Khongmee, Aranya from Chiang Mai University “*Escherichia coli* CRE10 isolated from stool of a human with gastroenteritis”, including *E. coli* CRE10 genome assembly GCA_004135815.1 and *mcr-1* plasmid assembly NZ_CP034405.1 are deposited in BioProject ID PRJNA508865 [112]. The software packages used in this study are free and open source. Scripts used to generate Dirichlet multinomial mixture models are included as Additional files 4, 5 and 6. Scripts used for functional metagenomics processing and analysis and scripts used for resistance gene annotation are available on GitHub at https://github.com/dantaslab [113].
