# Supplementary material for: Destination shapes antibiotic resistance gene acquisitions, abundance increases, and diversity changes in Dutch travelers
Source: Genome Med. 2021 Jun 7;13:79. doi: 10.1186/s13073-021-00893-z (PMC8182900; doi:10.1186/s13073-021-00893-z)
Supplement: Supplementary file 1 — Additional file 1: Supplementary notes (A-F), supplementary table legends (S1-S9), and supplementary figures (S1-S15). [file 13073_2021_893_MOESM1_ESM.pdf]

SUPPLEMENTARY NOTES, TABLE CAPTIONS, and FIGURES

Destination shapes antibiotic resistance gene acquisitions, abundance increases, and diversity changes in Dutch travelers.

Alaric W. D'Souza[1]*, Manish Boolchandani[1]*, Sanket Patel[1,2], Gianluca Galazzo[3], Jarne M. van Hattem[4], Maris S Arcilla[5], Damian C. Melles[5], Menno D. de Jong[4], Constance Schultsz[4,6], COMBAT consortium#, Gautam Dantas[1,2,7,8]**, John Penders[3,9]**

[1]The Edison Family Center for Genome Sciences and Systems Biology, Washington University School of Medicine, St. Louis, MO, USA

[2]Department of Pathology and Immunology, Washington University School of Medicine, St. Louis, MO, USA

[3]Department of Medical Microbiology, Care and Public Health Research Institute (Caphri), Maastricht University Medical Center, Maastricht, the Netherlands.

[4]Department of Medical Microbiology, Amsterdam University Medical Center, location AMC, Amsterdam, Netherlands.

[5]Department of Medical Microbiology and Infectious Diseases, Erasmus University Medical Centre, Rotterdam, Netherlands.

[6]Department of Global Health-Amsterdam-Institute for Global Health and Development, AMC, Amsterdam, the Netherlands.

[7]Department of Molecular Microbiology, Washington University School of Medicine, St. Louis, MO, USA

[8]Department of Biomedical Engineering, Washington University in St. Louis, St. Louis, MO, USA

[9]School for Nutrition and Translational Research in Metabolism (NUTRIM), Maastricht University Medical Center, Maastricht, the Netherlands

*These authors contributed equally to this work.

**Corresponding authors: (John Penders: j.penders@maastrichtuniversity.nl, Gautam Dantas: dantas@wustl.edu)

#COMBAT consortium

Martin C.J. Bootsma[10,11], Perry J. van Genderen[12], Abraham Goorhuis[13], Martin P. Grobusch[13], Nicky Molhoek[14], Astrid M.L. Oude Lashof[3], Ellen E. Stobberingh[3], Henri A. Verbrugh[5]

[10]Julius Centre for Health Sciences and Primary Care, University Medical Centre Utrecht, Utrecht, the Netherlands

[11]Department of Mathematics, Faculty of Science, Utrecht University, Utrecht, the Netherlands

[12]Department of Medical Microbiology and Infectious Diseases, University Hospital Erasmus Medical Centre, Rotterdam; Department of Internal Medicine and Institute for Tropical Diseases, Harbour Hospital Rotterdam

[13]Center of Tropical Medicine and Travel Medicine, Department of Infectious Diseases, Amsterdam University Medical Center, Amsterdam Public Health, Amsterdam Infection and Immunity, University of Amsterdam, Amsterdam, the Netherlands.

[14]Department of Internal Medicine and Institute for Tropical Diseases, Harbour Hospital, Rotterdam, The Netherlands.

SUPPLEMENTARY NOTES......4
A. What is a gene: Sequence Identity vs. Annotation......4
B. Resistome α-diversity is significantly higher after travel after correlation with abundance is accounted for. 4
C. Our results on the highest risk travel regions broadly agree with the AMR map published by the Center for Disease Dynamics, Economics, and Policy (CDDEP)......6
D. Subregion demonstrates resistome shaping effects better than continent. 7
E. Comparison to Indian resident gut resistomes......7
F. Resistance gene associations varied by timepoint, microbiota taxonomy, and destination. 8
References......11
SUPPLEMENTARY TABLE CAPTIONS......12
SUPPLEMENTARY FIGURES 13

SUPPLEMENTARY NOTES

A. What is a gene: Sequence Identity vs. Annotation

Since our ShortBRED marker sequences are built with both functionally selected genes and known databases, the categories for each gene can end up with high sequence identity ranges. We cluster our sequences at 95% sequence identity prior to building our marker sequences. These 95% sequence identity clusters correspond to our ShortBRED IDs. Though most identically annotated resistance genes are above this 95% threshold, not all meet this criterion. For ShortBRED ID, ShortBRED counts directly reflect abundance of input sequences.

B. Resistome α-diversity is significantly higher after travel after correlation with abundance is accounted for.

Since the abundance and α-diversity increases corresponded, we wanted to determine their relationship in the context of collection timepoint. To understand this relationship, we fitted linear mixed models (estimated using REML and nloptwrap optimizer) to predict Richness as the dependent variable (Additional file 2: Table S2). We then compared our different models together to understand the contribution of the different fixed effects (Additional file 2: Table S3). The results from our best model (shown in bold on Additional file 2: Table S2 and Additional file 2: Table S3) are described in Additional file 2: Table S4. This model had log(RPKM) and timepoint as interacting fixed effects. The model also included Subject_id as random effects. Standardized parameters were obtained by fitting the model on a standardized version of the dataset. Effect sizes were labeled following Funder et al. 2019 recommendations[1]. The model's total explanatory power is substantial (conditional $R^2 = 0.66$) and the part related to the fixed effects alone given by the marginal R^2 is 0.54. The model's intercept, corresponding to Richness = 0, RPKM = 0, Timepoint

= Pre-travel and Subject_id = R100241, is at -50.31 (SE = 6.60, 95% CI [-63.25, -37.36], $p<0.001$).

Within this model:

- The effect of log(RPKM) is positive and can be considered as very large and significant (beta = 13.35, SE = 0.86, 95% CI [11.66, 15.05], std. beta = 6.47, $p<0.001$).
- The effect of TimepointPost-travel is positive and can be considered as very large and significant (beta = 45.34, SE = 10.01, 95% CI [25.73, 64.96], std. beta = 4.63, $p<0.001$).
- The effect of log(RPKM):TimepointPost-travel is negative and can be considered as very large and significant (beta = -4.70, SE = 1.27, 95% CI [-7.19, -2.21], std. beta = -2.00, $p<0.001$).

The results from this model clearly show that both resistance gene abundance and travel contribute strongly to resistome α-diversity. However, though resistance gene abundance is a strong correlate, timepoint has a much larger effect size, indicating that travel is the major driver behind increases in resistome α-diversity.

Travel duration was the only other metadata variable given in Additional file 2: Table S1, with a significant effect on α-diversity. This effect is weak (see below) and including travel duration as an additional variable in the previous model does not improve the total explanatory power.

- The effect of log(RPKM) is positive and can be considered as very large and significant (beta = 13.17, SE = 0.86, 95% CI [11.49, 14.85], std. beta = 6.39, $p < .001$).
- The effect of TimepointPost-travel is positive and can be considered as very large and significant (beta = 44.87, SE = 9.95, 95% CI [25.36, 64.37], std. beta = 4.58, $p < .001$).
- The effect of Travel_duration is positive and can be considered as very small and significant (beta = 0.16, SE = 0.05, 95% CI [0.06, 0.26], std. beta = 0.12, $p < .01$).

- The effect of log(RPKM):TimepointPost-travel is negative and can be considered as very large and significant (beta = -4.63, SE = 1.26, 95% CI [-7.11, -2.15], std. beta = -1.97, $p < .001$).

C. Our results on the highest risk travel regions broadly agree with the AMR map published by the Center for Disease Dynamics, Economics, and Policy (CDDEP)

Tunisia was the only North African country on the CDDEP map [https://resistancemap.cddep.org/AntibioticResistance.php], and it has an AMR rate in *E. coli* of 19% against fluoroquinolones and 37% against 3rd gen cephalosporins with 78 isolates tested in 2017 (Additional file 2: Table S5). These were the lowest and second lowest AMR rates of any country tested from the subregion destinations represented in our cohort. Our analysis found that individuals returning from North Africa had the lowest AMR gene abundance increase, the lowest AMR gene α-diversity, the fewest AMR gene acquisitions, and the lowest mobile genetic element detection. Thus, our results from North Africa are consistent with the available data from the AMR map. By contrast the AMR rates in countries from the other three destination subregions were all much higher both in the AMR map (Additional file 2: Table S5) and in our results. However, the AMR rate in the Netherlands was even lower than the AMR rate in Tunisia by ~3% for fluoroquinolones and ~30% for 3rd gen cephalosporins (Additional file 2: Table S5) and these differences in AMR rate correlated with increased post-travel resistome abundance and diversity compared to the pre-travel controls. This comparison further highlights that endemic AMR in a country is correlated with the risk of AMR acquisition and resistome diversification in travelers visiting that region.

D. Subregion demonstrates resistome shaping effects better than continent.

If we look at continents instead of travel destinations, we see that Asia has lower β-diversity than Africa ($p=0.0016$ [unpaired wilcoxon test]) (Fig. S5A). Though, individuals going to the same continent had lower post-travel β-diversity than individuals going to different continents ($p=0.15$ [unpaired wilcoxon test]), this difference was not statistically significant (Fig. S5B). However, for individuals going to the same subregion, this β-diversity difference was significant ($p=0.016$ [unpaired wilcoxon test]) (Fig. S5C). This shows that subregions within the same continent do not necessarily act as dyads. The granular subregion level is valuable to understanding destination specific effects on the resistome.

E. Comparison to Indian resident gut resistomes

We profiled the resistomes of these Indian residents using the same ShortBRED database we used on our cohort. Next we found the pairwise Bray-Curtis dissimilarity of each sample in our cohort to each sample in the Indian resident cohort and we subtracted the pre-travel dissimilarity from the post-travel dissimilarity. This yielded the change in Bray-Curtis dissimilarity to the Indian residents before and after travel. Finally, we split our cohort into their subregion of travel. As we expected, we found that the pre-travel samples did not have much variability based on destination since they have not traveled yet. Interestingly when we look at change in dissimilarity from the Indian residents, we found that all of our destination groups moved further away from the Indian residents. This may be explained by the perturbation of travel having a strong effect even independent of destination. Despite this overall increase and despite differences in sequencing and extraction methods between the two cohorts, we found that individuals traveling to Southern Asia (which includes India) had resistomes that were most similar to the Indian resident's resistomes (Fig. S6).

F. Resistance gene associations varied by timepoint, microbiota taxonomy, and destination.

In the acquisition analysis, we looked resistance genes using presence or absence in the pre-travel and post-travel samples. To understand significant differences in abundance at the resistance gene level between the pre-travel and post-travel, we used MaAsLin2[2]. We conducted our analysis for resistance genes at the antibiotic class resistance determinant level, at the gene family level, and at the granular level provided by our 95% identity clustered ShortBRED IDs. MaAsLin2 uses linear models to perform multivariate associations between omics data like our resistome profiles and metadata variables like travel and travel destination. Importantly, MaAsLin2 can handle longitudinal data and account for random effects and multiple hypothesis testing. Thus, we built a model with our resistome profiles as the response variable and timepoint, travel destination, gut microbial taxonomy, and subject id as the input variables.

Using MetaPhlAn2, a taxonomic classifier, we identified 70 bacterial families within our gut microbiome samples[3]. To determine which taxonomic families to include in the model, we used a prevalence cutoff of 0.25 and a variance cutoff of 10 (Fig. S9). Eight taxonomic families passed these filtering thresholds and were included in the model.

At the resistance determinant level, we observed that 5 classes of resistance genes were significantly associated with the post-travel timepoint while only tetracycline resistance determinants ($p=9.47e-4$) were associated with the pre-travel timepoint (Fig. S10A). Of these, resistance determinants against trimethoprim ($p=1.07e-10$) were the most differential. Resistance determinants against sulfamethoxazole ($p=5.99e-7$) were also increased which is expected given the frequency of coformulation for these two drugs. When we look at destination using Northern Africa as our comparison group we see that the other three regions had several significantly increased resistance determinants (Fig. S10B). Resistance genes targeting antifolate drugs were

increased in every region compared to North Africa. Notably, determinants for β-lactam resistance and polymyxin resistance were not significant for either timepoint or destination at this grouping level. Analysis of these resistance determinants with the 8 taxonomic families showed that β-lactam resistance determinants all together only positively correlated with Prevotellaceae and polymyxin resistance determinants only positively correlated with Enterobacteriaceae (Fig. S10C). These results suggest that even at the high-level grouping for target drug affected by resistance determinants, there are significant correlates within our samples to timepoint, destination, and taxonomy.

At the gene family level, we have more power to distinguish within resistance classes and we observe that some β-lactam resistance gene families and some tetracycline resistance gene families are in fact significantly associated with the post-travel timepoint (Fig. S11A). For β-lactamases, we see that while a few class A and class C *bla* genes like bla_{cblA} (p=0.0356) and bla_{cfxA} (p=0.0164) are associated with before travel samples, others like bla_{TEM} (p=8.31e-10) are strongly associated with the post-travel timepoint. For tetracycline resistance genes, we see that while ribosomal protection proteins (p=3.28e-6) are strongly associated with the pre-travel timepoint, the other mechanisms of tetracycline resistance are associated with the post-travel timepoint. In both of these cases, it is likely that the results seen in (Fig. S11) were due to opposite effects from different gene families against the same antibiotics having opposed timepoint associations.

For destinations, we see some potential evidence of continent specific effects with the class A β-lactamases (Fig. S11B). Specifically, bla_{TEM} was significantly associated with Southern Asia (p=0.027) and Southeastern Asia (p=0.0041), but not with Eastern Africa. In contrast, unclassified class A β-lactamases were significantly associated with Eastern Africa (p=0.00141). This could

be some example of regional specificity within resistance determinants against specific antibiotic classes.

When we look at microbial taxonomy we see that the more granular gene family analysis gives similar insights (Fig. S12). Specifically, we now see significant β-lactamase genes associated with Enterobacteriaceae.

Of the 65 resistance genes significantly associated with time, 47 (72%) had significant positive association with the post-travel samples (Additional file 2: Table S6). This once again highlights the enrichment of resistance genes post-travel. The enriched genes included class A β-lactamases like *bla*$_{\mathrm{TEM}}$, *ampC* a class C β-lactamase, and trimethoprim-sulfamethoxazole genes like *dfrA* and *sul1*.

Associations with taxonomy were not as strong as the association with timepoint, but 101 resistance genes had a significant association with a bacterial taxon (Additional file 2: Table S6). Enterobacteriaceae had the most significantly correlated genes (n=65) with 37 positively correlated and 28 negatively correlated.

In the region-based analysis, only 5 genes were significantly positively correlated with Northern Africa compared to the other destinations (Additional file 2: Table S6). Not of the destination region correlated genes were positively correlated with Northern Africa compared to Eastern Africa. This trend agrees with the results from our previous analysis of overall resistance gene abundance by destination and of resistance gene acquisition by destination.

A model including all of the metadata variables from Additional file 2: Table S1 as fixed effects and Subject_ID and travel destination as random effects again confirmed that timepoint was the major predictor for most resistance genes. Several other metadata variables, including age, tap water consumption, antibiotic use, corticosteroid use, reason for traveling, raw vegetable

consumption, salad consumption, foodstall food consumption, hospitalization abroad, and main travel accommodation were found to have significant effects on a few resistance determinants (Additional file 2: Table S7). Notably, antibiotic use was associated with slight increase in class A β-lactamases and *aad9*, an aminoglycoside nucleotidyltransferase; travel duration was associated with an increase in *catA*, a chloramphenicol acetyltransferase.

References

1 Funder, D. C. & Ozer, D. J. Evaluating Effect Size in Psychological Research: Sense and Nonsense. *Advances in Methods and Practices in Psychological Science* **2**, 156-168, doi:10.1177/2515245919847202 (2019).

2 Mallick, H. *et al.* Multivariable Association in Population-scale Meta-omics Studies. *In submission* (2020).

3 Truong, D. T. *et al.* MetaPhlAn2 for enhanced metagenomic taxonomic profiling. *Nat Methods* **12**, 902-903, doi:10.1038/nmeth.3589 (2015).

SUPPLEMENTARY TABLE CAPTIONS

Table S1: Netherland traveler cohort metadata

Table S2: Model formulas for resistome α-diversity as a function of AMR gene abundance

Table S3: Model performance statistics for resistome α-diversity as a function of AMR gene abundance

Table S4: Results from best performing (interaction) model of resistome α-diversity as a function of AMR gene abundance

Table S5: *E. coli* resistance data for fluoroquinolones and 3rd gen cephalosporins from Resmap [https://resistancemap.cddep.org/AntibioticResistance.php] by the Center for Disease Dynamics, Economics, and Policy. Data from countries in our 4 cohort subregion destinations was included. To generate these data, bacterial isolates gathered in each country are tested for AMR against antibiotics using standardized AMR breakpoints [https://resistancemap.cddep.org/Methodology.php].

Table S6: Results from MaAsLin2 model for AMR genes significantly associated with timepoint, travel destination, and 8 prevalent taxonomic families.

Table S7: Results from MaAsLin2 model for AMR gene abundance including all cohort metadata variables from Table S1 as predictor variables and Subject_ID and travel destination as random effects. Source data for this model is available in the source data file.

Table S8: ShortBRED IDs and metadata for genes detected in Netherland traveler stool samples.

Table S9: Antibiotics concentrations used in functional metagenomics screening. The antibiotic selections were performed in Mueller-Hinton agar with 50 μg/ml kanamycin.

SUPPLEMENTARY FIGURES

Fig. S1: Functional metagenomics and ShortBRED database workflow figure.

21 functional metagenomics libraries were built using cohort samples with selections against 15 antibiotics. The resultant reads were assembled and annotated. AMR genes were then used to build a ShortBRED marker database.

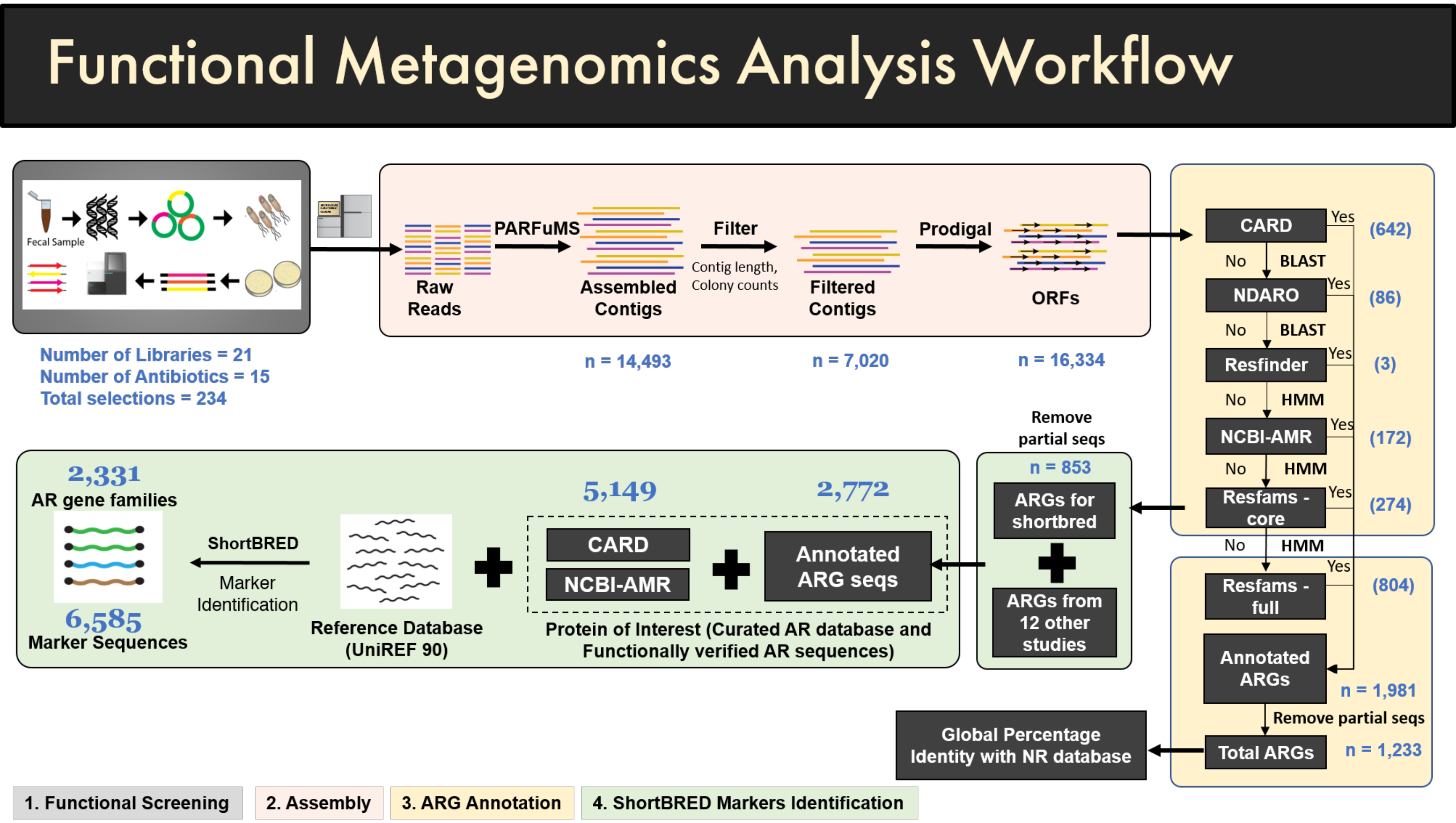
Functional Metagenomics Analysis Workflow
Fecal Sample
Raw Reads
PARFuMS
Assembled Contigs
Filter
Contig length, Colony counts
Filtered Contigs
Prodigal
ORFs
Number of Libraries = 21
Number of Antibiotics = 15
Total selections = 234
n = 14,493
n = 7,020
n = 16,334
CARD
Yes
(642)
No
BLAST
NDARO
Yes
(86)
No
BLAST
Resfinder
Yes
(3)
No
HMM
NCBI-AMR
Yes
(172)
No
HMM
Resfams - core
Yes
(274)
No
HMM
Resfams - full
Yes
(804)
Annotated ARGs
n = 1,981
Remove partial seqs
Total ARGs
n = 1,233
Global Percentage Identity with NR database
Remove partial seqs
n = 853
ARGs for shortbred
ARGs from 12 other studies
2,331
AR gene families
ShortBRED
Marker Identification
6,585
Marker Sequences
Reference Database (UniREF 90)
5,149
2,772
CARD
NCBI-AMR
Annotated ARG seqs
Protein of Interest (Curated AR database and Functionally verified AR sequences)
1. Functional Screening
2. Assembly
3. ARG Annotation
4. ShortBRED Markers Identification

Fig. S2: Post-travel associated metaresistomes' timepoints frequently have higher α-diversity and lower β-diversity

The top panel shows α-diversity (Shannon Index) measurements for the 8 metaresistomes defined in Main manuscript file: Fig. 3. Boxes are filled according to which timepoint metaresistomes were significantly associated with (blue for pre-travel, red for post-travel, and black for neither). The bottom panel shows the Bray-Curtis dissimilarity between metaresistomes. The columns (x-axis) gives the reference group and the colored text on the plot gives the comparison group. The y-axis position gives the β-diversity between the reference and comparison groups. All text is colored according to timepoint association (blue for pre-travel, red for post-travel, and black for neither). Source data for all panels is provided in the source data file.

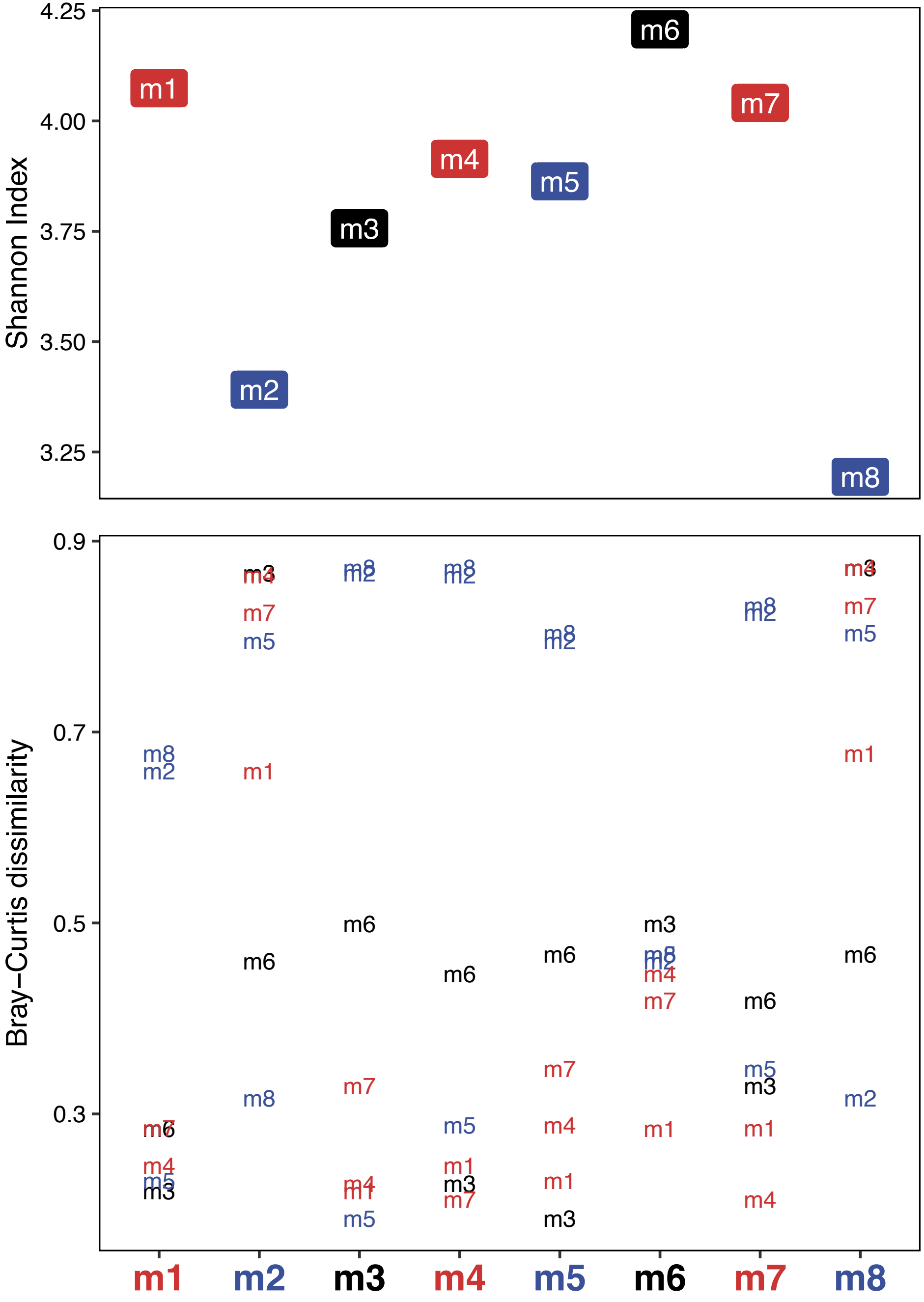

Shannon Index
4.25
4.00
3.75
3.50
3.25
Bray–Curtis dissimilarity
0.9
0.7
0.5
0.3
m1
m2
m3
m4
m5
m6
m7
m8

Fig. S3: Supervised clustering shows separation by timepoint and destination

A Capscale ordination by timepoint. Each point on the graph is a single sample and each line on the graph connects a sample point to the centroid of the cluster for that sample's timepoint. Blue points, lines, and labels are for pre-travel samples, and red points, lines, and labels are for post-travel samples. The p-value (permanova) is given at the top of the figure.

Capscale ordination by destination for pre- (**B**) and post- (**C**) travel samples. Each point on the graph is a single sample and each line on the graph connects a sample point to the centroid of the cluster for that sample's destination. For lines, points, and labels, color corresponds to destination region (Dark blue is Northern Africa, light blue is Eastern Africa, orange is Southern Asia, and red is Southeastern Asia). P-values (permanova) are given in the top left of each plot. Source data for all panels is provided in the source data file.

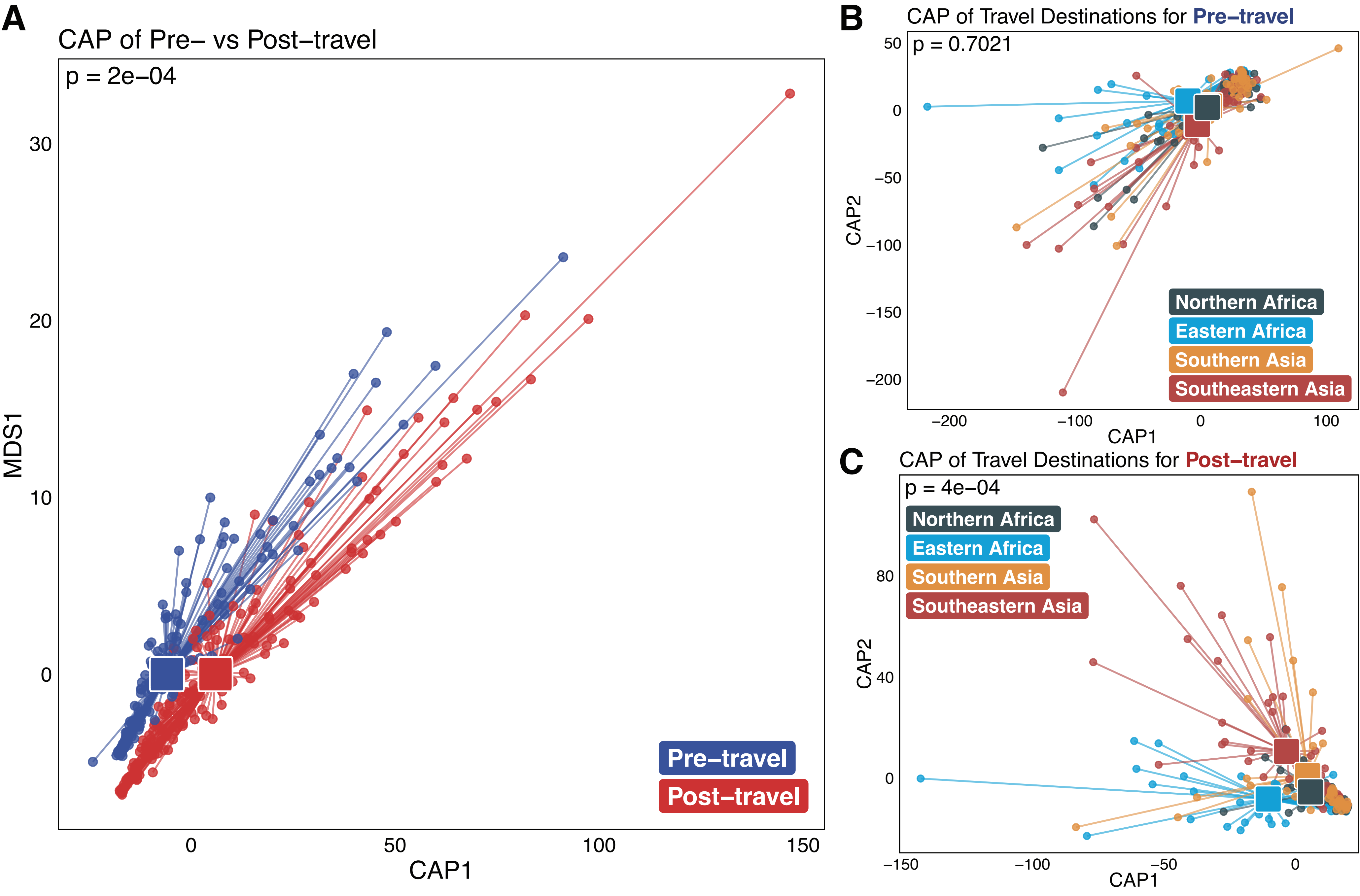
A
CAP of Pre– vs Post–travel
p = 2e–04
MDS1
CAP1
Pre–travel
Post–travel
B
CAP of Travel Destinations for Pre–travel
p = 0.7021
CAP2
CAP1
C
CAP of Travel Destinations for Post–travel
p = 4e–04
CAP2
CAP1
Northern Africa
Eastern Africa
Southern Asia
Southeastern Asia

Fig. S4: Travelers to Southeastern Asia has higher post-travel resistome α-diversity

A Tukey's range test of post-travel resistome richness by travel destination showing 95% family-wise confidence level (lines) and mean difference (points) for all pairwise destination comparisons. Multiple hypothesis corrected p-values are given in line with each comparison. Significant comparisons are highlighted in red.

B Post-travel resistome richness for all travel destinations where each point is an individual sample. Boxes give the median and interquartile ranges.

Source data for all panels is provided in the source data file.

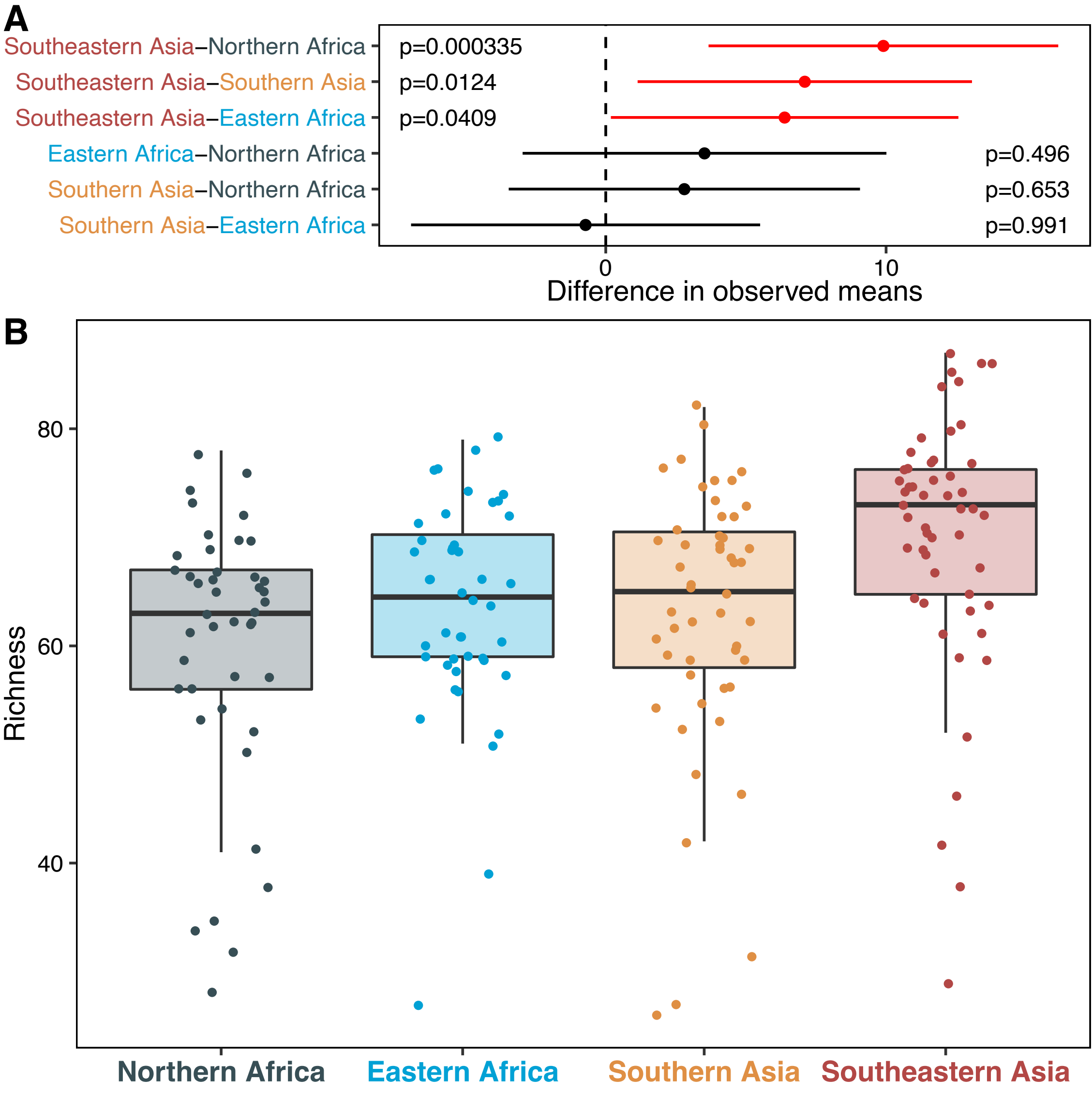
A
Southeastern Asia–Northern Africa
Southeastern Asia–Southern Asia
Southeastern Asia–Eastern Africa
Eastern Africa–Northern Africa
Southern Asia–Northern Africa
Southern Asia–Eastern Africa
p=0.000335
p=0.0124
p=0.0409
p=0.496
p=0.653
p=0.991
0
10
Difference in observed means
B
Richness
80
60
40
Northern Africa
Eastern Africa
Southern Asia
Southeastern Asia

Fig. S5: Resistome β-diversity was significantly lower for individuals traveling to the same Subregion, but not for individuals traveling to the same continent.

β-diversity comparisons between **A** Africa (blue) and Asia (red-orange), **B** same continent (gray) or different continents (green), **C** same destination subregion (gray) or different destination subregions (green). Each point is a pairwise Bray-Curtis dissimilarity between two post-travel samples and the boxes represent the median and interquartile ranges. The distributions are visualized to the right of the points. P-values (unpaired wilcoxon test) are given near the top of each plot. Source data for all panels is provided in the source data file.

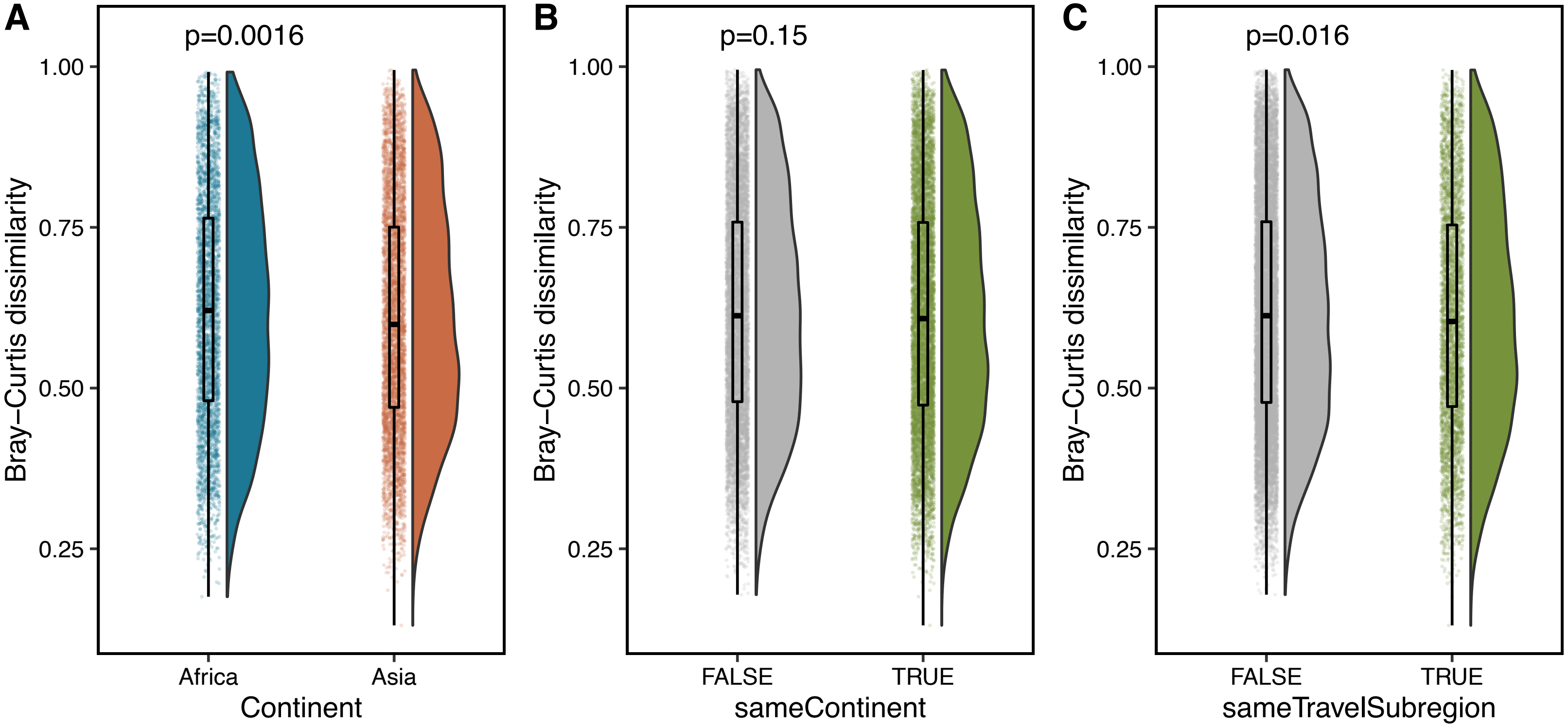
A
p=0.0016
Bray−Curtis dissimilarity
1.00
0.75
0.50
0.25
Africa
Asia
Continent
B
p=0.15
Bray−Curtis dissimilarity
1.00
0.75
0.50
0.25
FALSE
TRUE
sameContinent
C
p=0.016
Bray−Curtis dissimilarity
1.00
0.75
0.50
0.25
FALSE
TRUE
sameTravelSubregion

Fig. S6: Indian residents' resistomes were more similar to travelers to Southern Asia than to travelers to other regions.

A Difference in Bray-Curtis dissimilarity between post- and pre-travel samples by region. Each point is the difference of two pairwise comparisons between a Dutch traveler and an Indian resident. The boxplots give the median and interquartile range for each distribution and the shaded region gives depicts the distribution density. P-values by fdr corrected unpaired wilcoxon test are given above. **B** The lines are the 95% confidence intervals and points are the estimates for the distributions shown in panel A. The dotted black line shows the null hypothesis of no change. Source data for all panels is provided in the source data file.

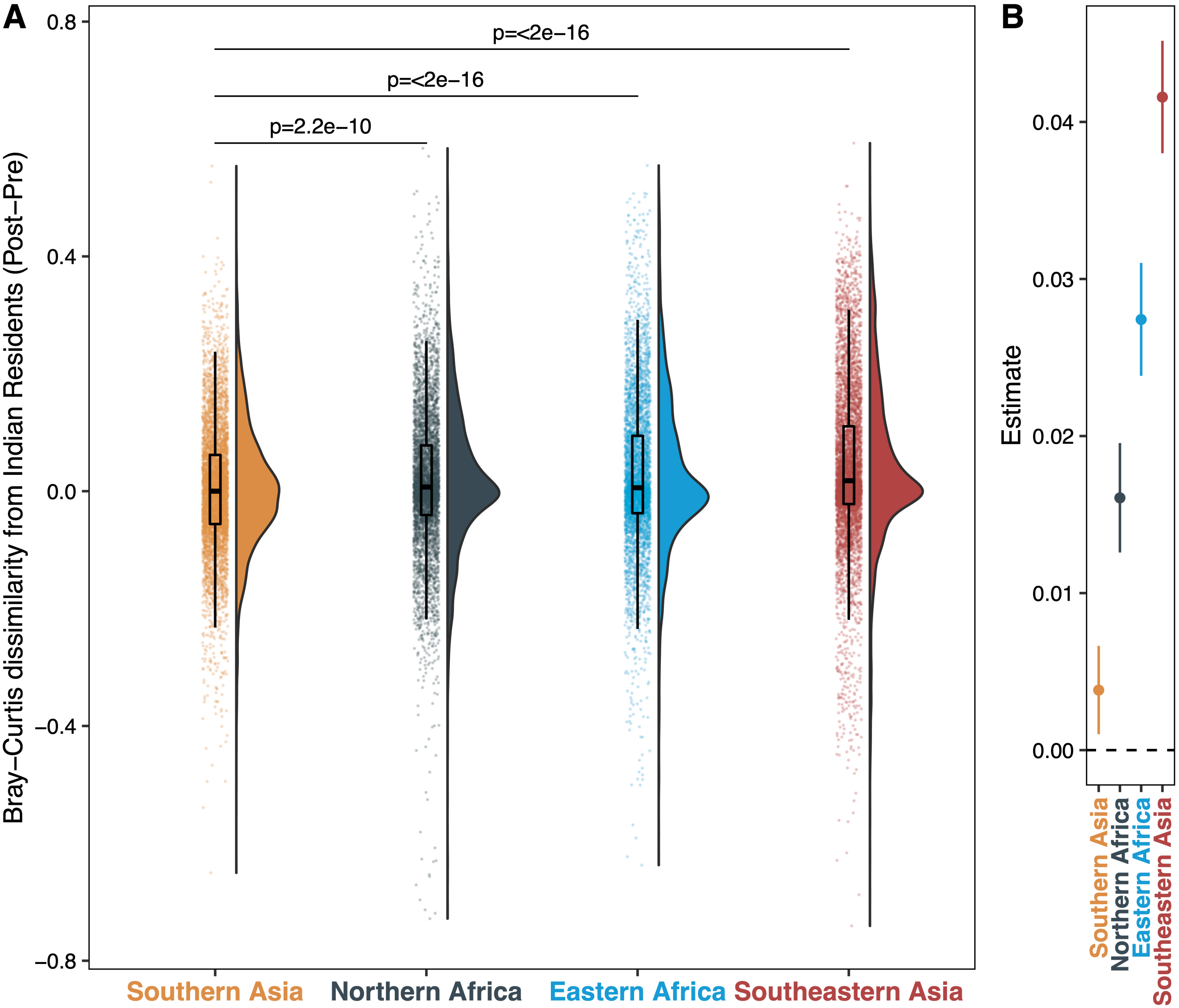
A
B
Bray–Curtis dissimilarity from Indian Residents (Post–Pre)
0.8
0.4
0.0
−0.4
−0.8
p=<2e−16
p=<2e−16
p=2.2e−10
Southern Asia
Northern Africa
Eastern Africa
Southeastern Asia
Estimate
0.04
0.03
0.02
0.01
0.00
Southern Asia
Northern Africa
Eastern Africa
Southeastern Asia

Fig. S7: Relationship between AMR gene prevalence and abundance varies by AMR mechanism.

The top left panel shows the relationship between AMR gene prevalence (x-axis) and AMR gene abundance (y-axis). The colors of the points correspond to the mechanism of AMR for the gene represented by the point. The exploded panels on the bottom right show this same relationship for the AMR mechanisms considered individually. In these panels, the colors further subdivide the AMR mechanisms into AMR class. In all panels, the black line is the best fit linear trendline through the points and the gray shaded region is the 95% confidence interval for this trendline. The fdr corrected p-value for the relationship is given in the top right of each panel. Source data for all panels is provided in the source data file.

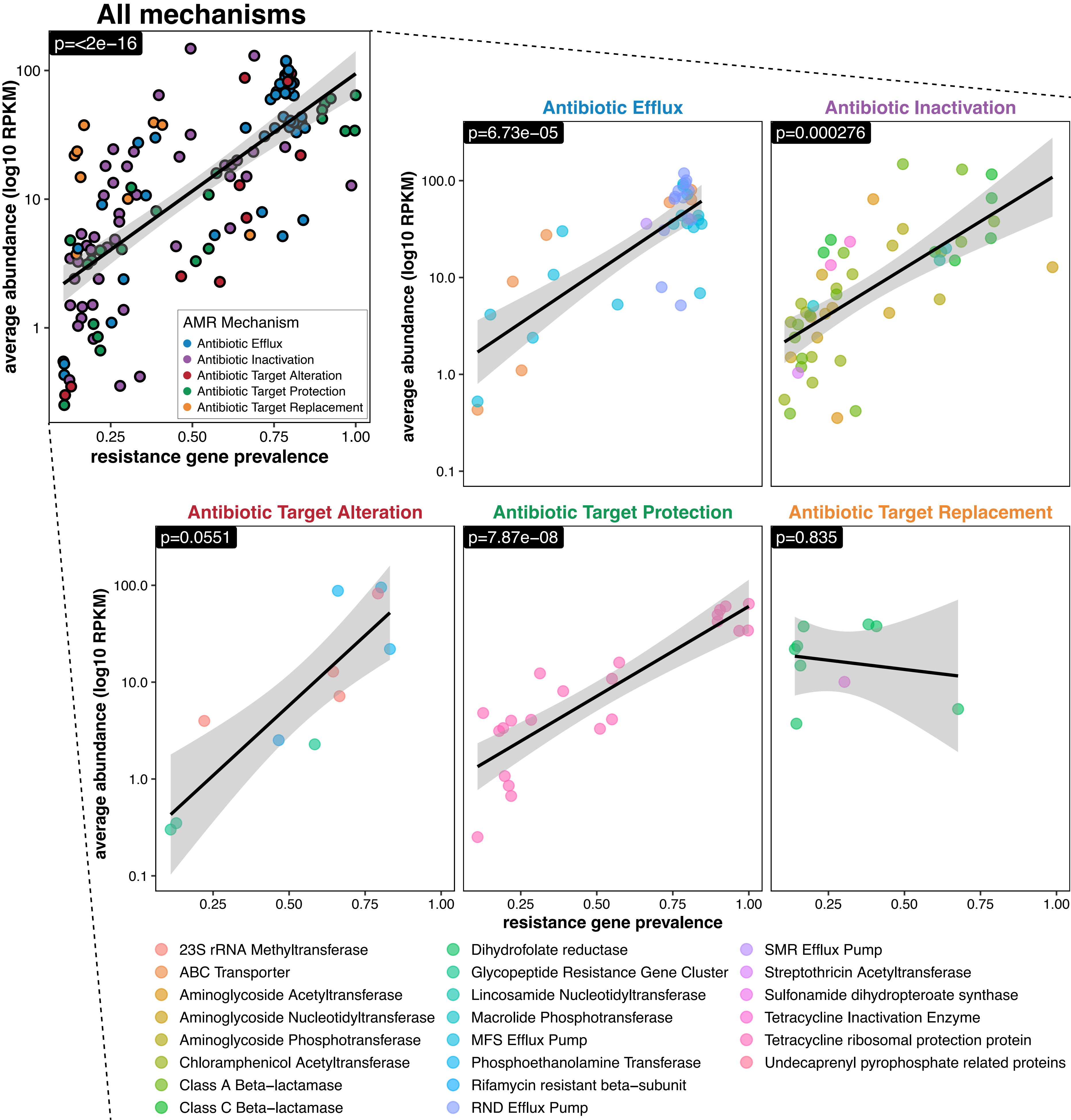

All mechanisms
p=<2e−16
average abundance (log10 RPKM)
resistance gene prevalence
AMR Mechanism
Antibiotic Efflux
Antibiotic Inactivation
Antibiotic Target Alteration
Antibiotic Target Protection
Antibiotic Target Replacement
Antibiotic Efflux
p=6.73e−05
Antibiotic Inactivation
p=0.000276
Antibiotic Target Alteration
p=0.0551
Antibiotic Target Protection
p=7.87e−08
Antibiotic Target Replacement
p=0.835
23S rRNA Methyltransferase
ABC Transporter
Aminoglycoside Acetyltransferase
Aminoglycoside Nucleotidyltransferase
Aminoglycoside Phosphotransferase
Chloramphenicol Acetyltransferase
Class A Beta−lactamase
Class C Beta−lactamase
Dihydrofolate reductase
Glycopeptide Resistance Gene Cluster
Lincosamide Nucleotidyltransferase
Macrolide Phosphotransferase
MFS Efflux Pump
Phosphoethanolamine Transferase
Rifamycin resistant beta−subunit
RND Efflux Pump
SMR Efflux Pump
Streptothricin Acetyltransferase
Sulfonamide dihydropteroate synthase
Tetracycline Inactivation Enzyme
Tetracycline ribosomal protection protein
Undecaprenyl pyrophosphate related proteins

Fig. S8: Most AMR genes are acquired during travel.

The results from binomial tests of bias for AMR gene ShortBRED ID acquisition for the post-travel timepoint. Lines are 95% confidence intervals and points are estimates. P-values (fdr corrected binomial test) are given at the bottom of the plot for each gene. The dotted line is the expected value under the null. Lines and points are red if significantly acquired and blue if significantly lost. Source data is provided in the source data file.

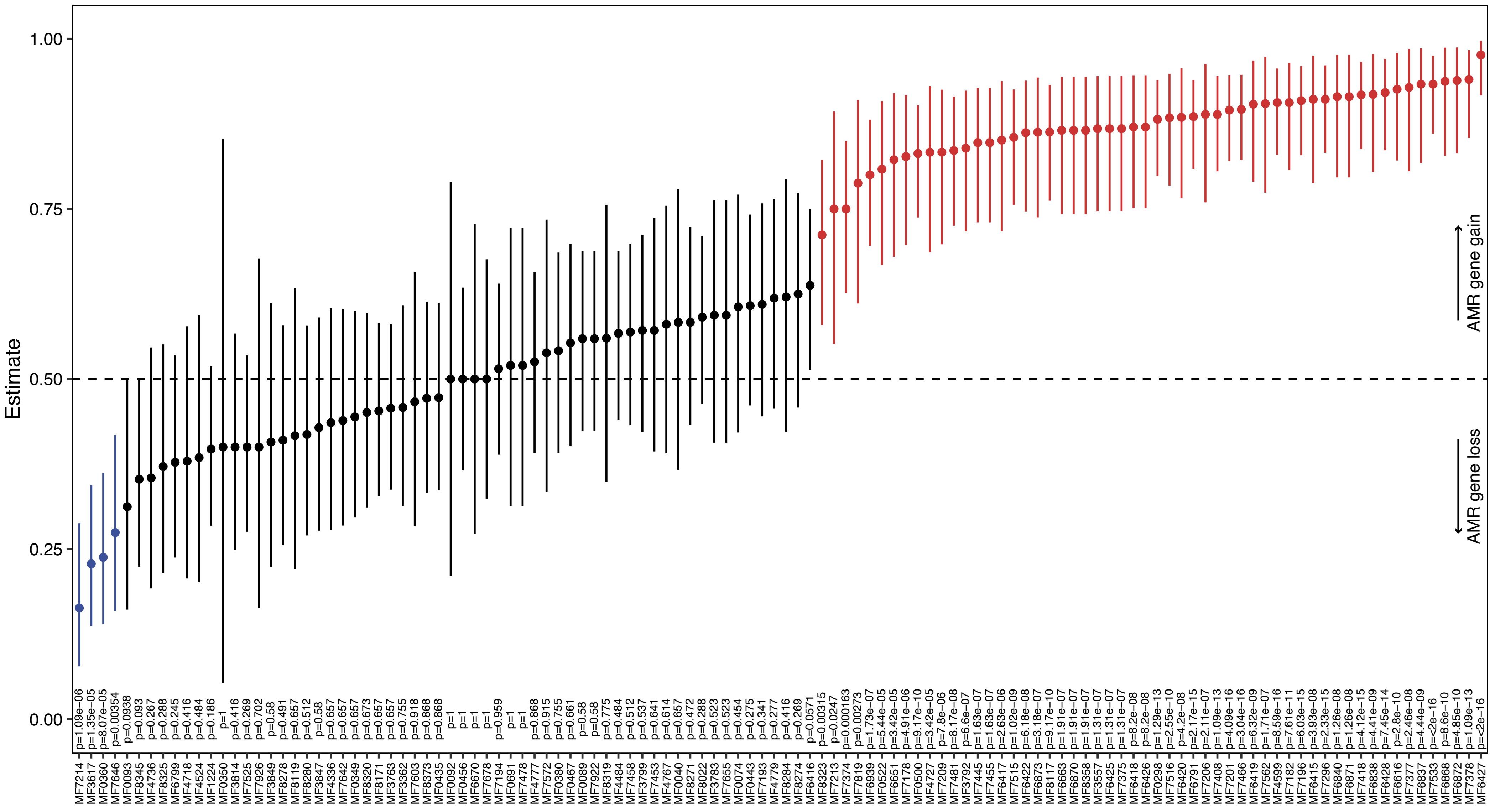

Estimate
1.00
0.75
0.50
0.25
0.00
AMR gene gain
AMR gene loss
p=1.09e−06
p=1.35e−05
p=8.07e−05
p=0.00354
p=0.0938
p=0.093
p=0.267
p=0.288
p=0.245
p=0.416
p=0.484
p=0.186
p=1
p=0.416
p=0.269
p=0.702
p=0.58
p=0.491
p=0.657
p=0.512
p=0.58
p=0.657
p=0.657
p=0.657
p=0.673
p=0.657
p=0.657
p=0.755
p=0.918
p=0.868
p=0.868
p=1
p=1
p=1
p=1
p=0.959
p=1
p=1
p=0.868
p=0.915
p=0.755
p=0.661
p=0.58
p=0.58
p=0.775
p=0.484
p=0.512
p=0.537
p=0.641
p=0.614
p=0.657
p=0.472
p=0.288
p=0.523
p=0.523
p=0.454
p=0.275
p=0.341
p=0.277
p=0.416
p=0.269
p=0.0571
p=0.00315
p=0.0247
p=0.000163
p=0.00273
p=1.73e−07
p=5.44e−05
p=3.42e−05
p=4.91e−06
p=9.17e−10
p=3.42e−05
p=7.8e−06
p=8.17e−08
p=6.6e−07
p=1.63e−07
p=1.63e−07
p=2.63e−06
p=1.02e−09
p=6.18e−08
p=3.18e−07
p=9.17e−10
p=1.91e−07
p=1.91e−07
p=1.91e−07
p=1.31e−07
p=1.31e−07
p=1.31e−07
p=8.2e−08
p=8.2e−08
p=1.29e−13
p=2.55e−10
p=4.2e−08
p=2.17e−15
p=2.11e−07
p=1.09e−13
p=4.09e−16
p=3.04e−16
p=6.32e−09
p=1.71e−07
p=8.59e−16
p=7.61e−11
p=6.03e−15
p=3.93e−08
p=2.33e−15
p=1.26e−08
p=1.26e−08
p=4.12e−15
p=4.41e−09
p=7.45e−14
p=2.8e−10
p=2.46e−08
p=4.44e−09
p=<2e−16
p=8.6e−10
p=4.85e−10
p=1.09e−13
p=<2e−16
MF7214
MF3617
MF0360
MF7646
MF0093
MF8345
MF4736
MF8325
MF6799
MF4718
MF4524
MF1224
MF0350
MF3814
MF7525
MF7926
MF3849
MF8278
MF8119
MF8280
MF3847
MF4336
MF7642
MF0349
MF8320
MF8171
MF3763
MF3362
MF7603
MF8373
MF0435
MF0092
MF0456
MF6670
MF7678
MF7194
MF0691
MF7478
MF4777
MF7572
MF0380
MF0467
MF0089
MF7922
MF8319
MF4484
MF7458
MF3799
MF7453
MF4767
MF0040
MF8271
MF8022
MF3783
MF7655
MF0074
MF0443
MF7193
MF4779
MF8284
MF8274
MF6416
MF8323
MF7213
MF7374
MF7819
MF6939
MF0522
MF6651
MF7178
MF0500
MF4727
MF7209
MF7481
MF3792
MF7445
MF7455
MF6417
MF7515
MF6422
MF6873
MF8117
MF6663
MF6870
MF8358
MF3557
MF6425
MF7375
MF6418
MF6426
MF0298
MF7516
MF6420
MF6791
MF7206
MF7408
MF7201
MF7466
MF6419
MF7562
MF4599
MF7182
MF7196
MF6415
MF7296
MF6840
MF6871
MF7418
MF6838
MF6428
MF6616
MF7377
MF6837
MF7533
MF6868
MF6872
MF7378
MF6427

Fig. S9: Only eight bacterial taxa were prevalent with high variance.

The x-axis is prevalence and the y-axis is the square root of variance. Dotted lines are the prevalence and variance cutoffs for inclusion in the MaAsLin2 model. Points on the graph are bacterial families that did not meet both of the cutoffs. Families that did meet the cutoff are in red labels. Source data is provided in the source data file.

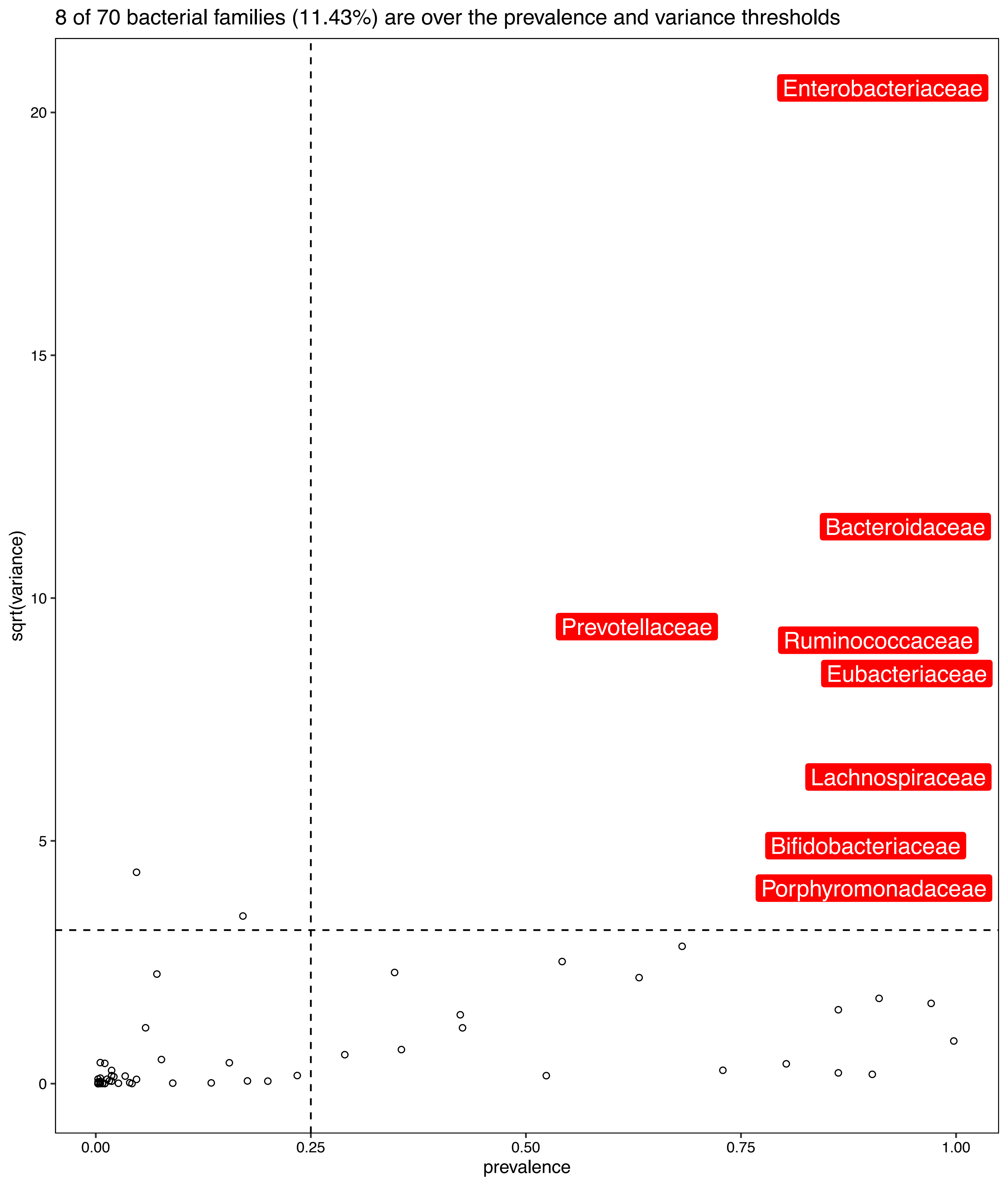
8 of 70 bacterial families (11.43%) are over the prevalence and variance thresholds
Enterobacteriaceae
Bacteroidaceae
Prevotellaceae
Ruminococcaceae
Eubacteriaceae
Lachnospiraceae
Bifidobacteriaceae
Porphyromonadaceae
sqrt(variance)
prevalence
0
5
10
15
20
0.00
0.25
0.50
0.75
1.00

Fig. S10: AMR determinants are enriched after travel.

A left panel is log-transformed abundance of significant AMR determinants in pre-travel (red) and post-travel (blue). Each point is the abundance of the AMR gene in one sample. Boxplots show the medians and interquartile ranges for these distributions. The right panel gives the model coefficients for these AMR determinants. **B** model coefficients for AMR determinants significantly associated with each subregion are shown. North Africa was the reference group. **C** model coefficients for AMR determinants significantly associated with taxonomic families are shown. In coefficient plots, bars are the coefficients and black lines are the standard deviation. Source data is provided in the source data file.

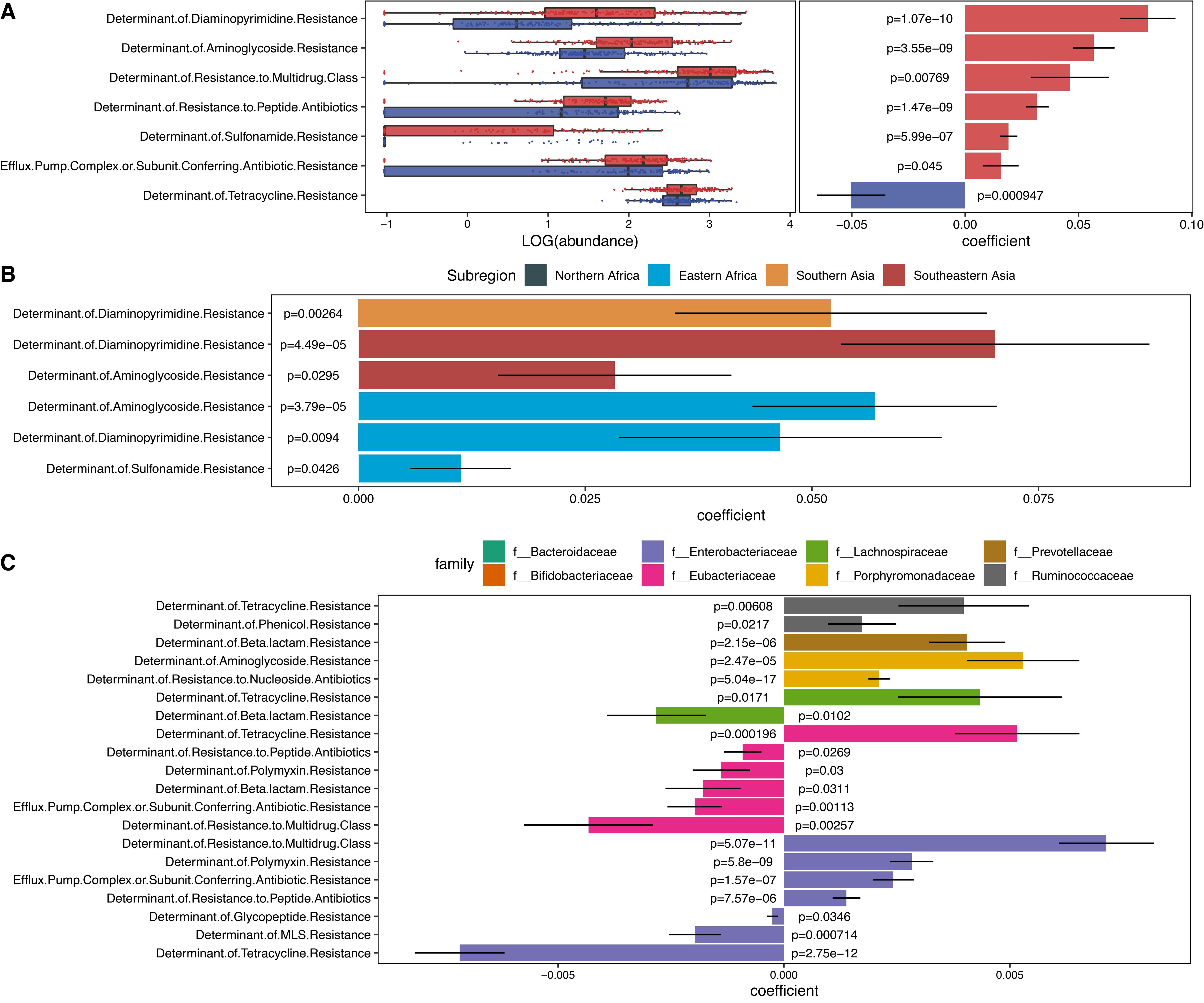
A
Determinant.of.Diaminopyrimidine.Resistance
Determinant.of.Aminoglycoside.Resistance
Determinant.of.Resistance.to.Multidrug.Class
Determinant.of.Resistance.to.Peptide.Antibiotics
Determinant.of.Sulfonamide.Resistance
Efflux.Pump.Complex.or.Subunit.Conferring.Antibiotic.Resistance
Determinant.of.Tetracycline.Resistance
−1 0 1 2 3 4
LOG(abundance)
p=1.07e−10
p=3.55e−09
p=0.00769
p=1.47e−09
p=5.99e−07
p=0.045
p=0.000947
−0.05 0.00 0.05 0.10
coefficient
B
Subregion
Northern Africa
Eastern Africa
Southern Asia
Southeastern Asia
Determinant.of.Diaminopyrimidine.Resistance p=0.00264
Determinant.of.Diaminopyrimidine.Resistance p=4.49e−05
Determinant.of.Aminoglycoside.Resistance p=0.0295
Determinant.of.Aminoglycoside.Resistance p=3.79e−05
Determinant.of.Diaminopyrimidine.Resistance p=0.0094
Determinant.of.Sulfonamide.Resistance p=0.0426
0.000 0.025 0.050 0.075
coefficient
C
family
f__Bacteroidaceae
f__Bifidobacteriaceae
f__Enterobacteriaceae
f__Eubacteriaceae
f__Lachnospiraceae
f__Porphyromonadaceae
f__Prevotellaceae
f__Ruminococcaceae
Determinant.of.Tetracycline.Resistance p=0.00608
Determinant.of.Phenicol.Resistance p=0.0217
Determinant.of.Beta.lactam.Resistance p=2.15e−06
Determinant.of.Aminoglycoside.Resistance p=2.47e−05
Determinant.of.Resistance.to.Nucleoside.Antibiotics p=5.04e−17
Determinant.of.Tetracycline.Resistance p=0.0171
Determinant.of.Beta.lactam.Resistance p=0.0102
Determinant.of.Tetracycline.Resistance p=0.000196
Determinant.of.Resistance.to.Peptide.Antibiotics p=0.0269
Determinant.of.Polymyxin.Resistance p=0.03
Determinant.of.Beta.lactam.Resistance p=0.0311
Efflux.Pump.Complex.or.Subunit.Conferring.Antibiotic.Resistance p=0.00113
Determinant.of.Resistance.to.Multidrug.Class p=0.00257
Determinant.of.Resistance.to.Multidrug.Class p=5.07e−11
Determinant.of.Polymyxin.Resistance p=5.8e−09
Efflux.Pump.Complex.or.Subunit.Conferring.Antibiotic.Resistance p=1.57e−07
Determinant.of.Resistance.to.Peptide.Antibiotics p=7.57e−06
Determinant.of.Glycopeptide.Resistance p=0.0346
Determinant.of.MLS.Resistance p=0.000714
Determinant.of.Tetracycline.Resistance p=2.75e−12
−0.005 0.000 0.005
coefficient

Fig. S11: AMR gene families are enriched after travel.

A left panel is log-transformed abundance of significant AMR gene families in pre-travel (red) and post-travel (blue). Each point is the abundance of the AMR gene in one sample. Boxplots show the medians and interquartile ranges for these distributions. The right panel gives the model coefficients for these AMR determinants. **B** model coefficients for AMR gene families significantly associated with each subregion are shown. North Africa was the reference group. In coefficient plots, bars are the coefficients and black lines are the standard deviation. Source data is provided in the source data file.

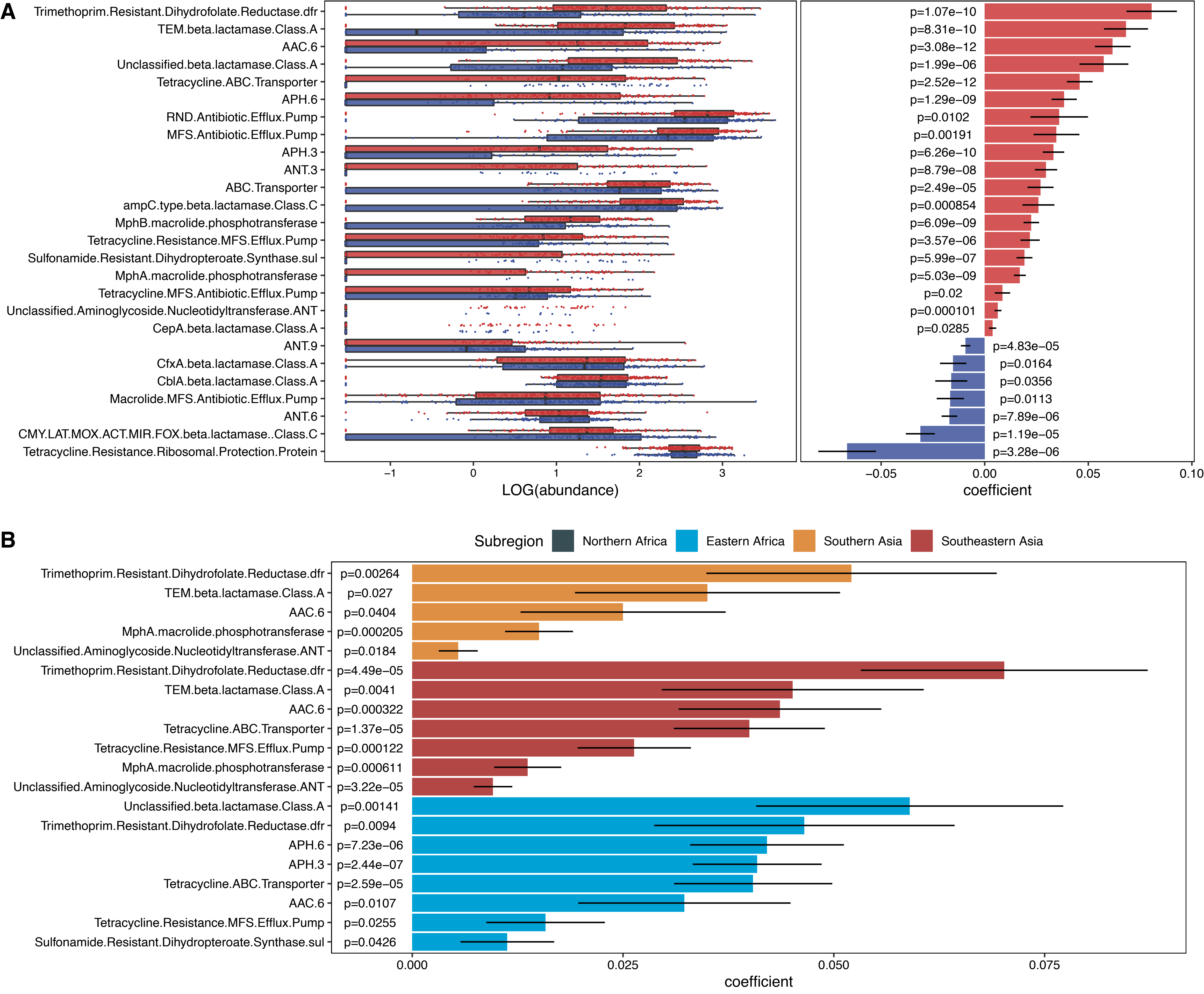
A
Trimethoprim.Resistant.Dihydrofolate.Reductase.dfr
TEM.beta.lactamase.Class.A
AAC.6
Unclassified.beta.lactamase.Class.A
Tetracycline.ABC.Transporter
APH.6
RND.Antibiotic.Efflux.Pump
MFS.Antibiotic.Efflux.Pump
APH.3
ANT.3
ABC.Transporter
ampC.type.beta.lactamase.Class.C
MphB.macrolide.phosphotransferase
Tetracycline.Resistance.MFS.Efflux.Pump
Sulfonamide.Resistant.Dihydropteroate.Synthase.sul
MphA.macrolide.phosphotransferase
Tetracycline.MFS.Antibiotic.Efflux.Pump
Unclassified.Aminoglycoside.Nucleotidyltransferase.ANT
CepA.beta.lactamase.Class.A
ANT.9
CfxA.beta.lactamase.Class.A
CblA.beta.lactamase.Class.A
Macrolide.MFS.Antibiotic.Efflux.Pump
ANT.6
CMY.LAT.MOX.ACT.MIR.FOX.beta.lactamase..Class.C
Tetracycline.Resistance.Ribosomal.Protection.Protein
−1
0
1
2
3
LOG(abundance)
p=1.07e−10
p=8.31e−10
p=3.08e−12
p=1.99e−06
p=2.52e−12
p=1.29e−09
p=0.0102
p=0.00191
p=6.26e−10
p=8.79e−08
p=2.49e−05
p=0.000854
p=6.09e−09
p=3.57e−06
p=5.99e−07
p=5.03e−09
p=0.02
p=0.000101
p=0.0285
p=4.83e−05
p=0.0164
p=0.0356
p=0.0113
p=7.89e−06
p=1.19e−05
p=3.28e−06
−0.05
0.00
0.05
0.10
coefficient
B
Subregion
Northern Africa
Eastern Africa
Southern Asia
Southeastern Asia
Trimethoprim.Resistant.Dihydrofolate.Reductase.dfr p=0.00264
TEM.beta.lactamase.Class.A p=0.027
AAC.6 p=0.0404
MphA.macrolide.phosphotransferase p=0.000205
Unclassified.Aminoglycoside.Nucleotidyltransferase.ANT p=0.0184
Trimethoprim.Resistant.Dihydrofolate.Reductase.dfr p=4.49e−05
TEM.beta.lactamase.Class.A p=0.0041
AAC.6 p=0.000322
Tetracycline.ABC.Transporter p=1.37e−05
Tetracycline.Resistance.MFS.Efflux.Pump p=0.000122
MphA.macrolide.phosphotransferase p=0.000611
Unclassified.Aminoglycoside.Nucleotidyltransferase.ANT p=3.22e−05
Unclassified.beta.lactamase.Class.A p=0.00141
Trimethoprim.Resistant.Dihydrofolate.Reductase.dfr p=0.0094
APH.6 p=7.23e−06
APH.3 p=2.44e−07
Tetracycline.ABC.Transporter p=2.59e−05
AAC.6 p=0.0107
Tetracycline.Resistance.MFS.Efflux.Pump p=0.0255
Sulfonamide.Resistant.Dihydropteroate.Synthase.sul p=0.0426
0.000
0.025
0.050
0.075
coefficient

Fig. S12: Some AMR gene families are taxonomically linked.

Model coefficients for AMR gene families significantly associated with taxonomic families are shown. Bars are the coefficients and black lines are the standard deviation. The p-values for each association is above or below the coefficient. Source data is provided in the source data file.

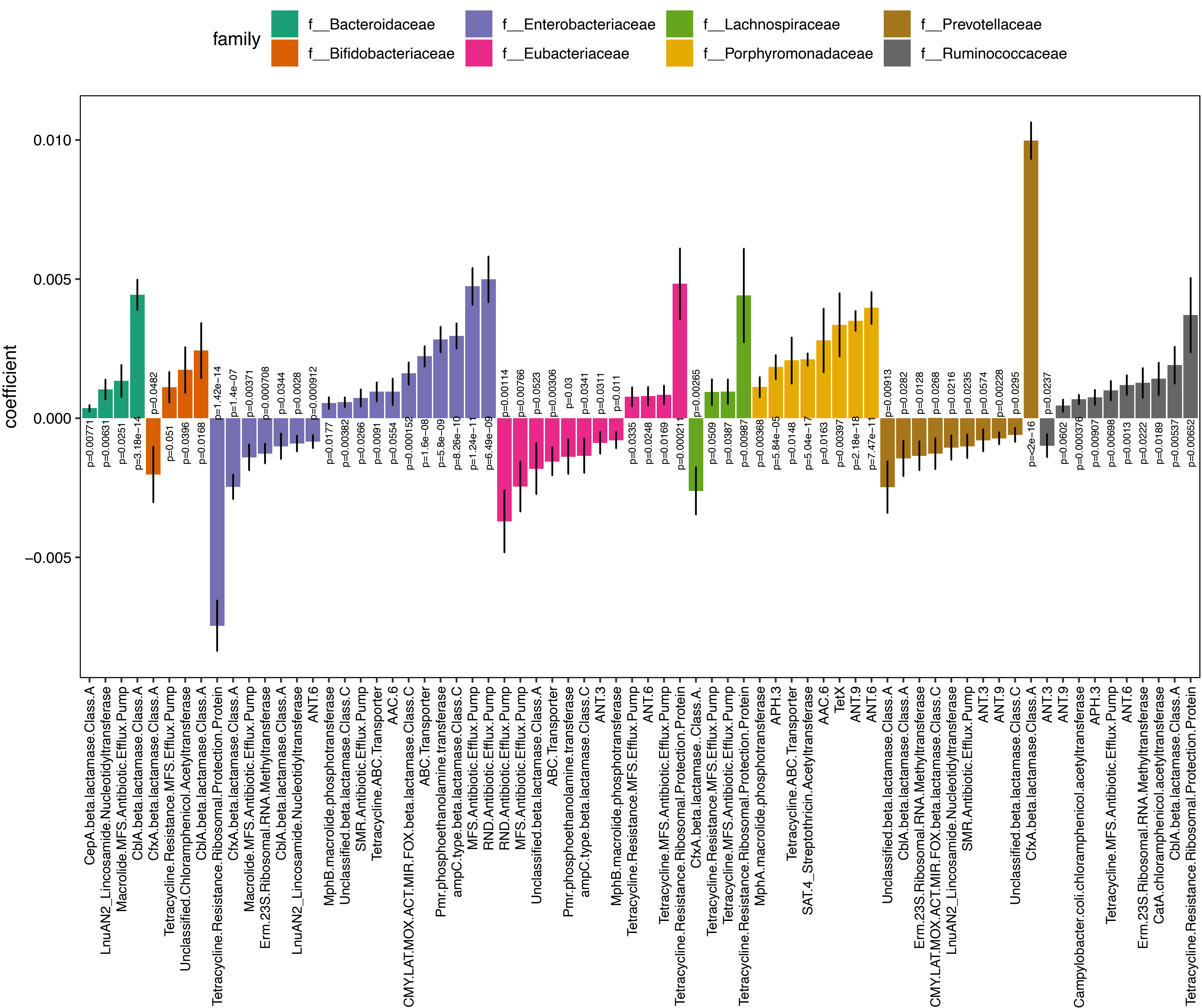

family
f__Bacteroidaceae
f__Bifidobacteriaceae
f__Enterobacteriaceae
f__Eubacteriaceae
f__Lachnospiraceae
f__Porphyromonadaceae
f__Prevotellaceae
f__Ruminococcaceae
coefficient
0.010
0.005
0.000
-0.005
CepA.beta.lactamase.Class.A p=0.00771
LnuAN2_Lincosamide.Nucleotidyltransferase p=0.00631
Macrolide.MFS.Antibiotic.Efflux.Pump p=0.0251
CblA.beta.lactamase.Class.A p=3.18e−14
CfxA.beta.lactamase.Class.A p=0.0482
Tetracycline.Resistance.MFS.Efflux.Pump p=0.051
Unclassified.Chloramphenicol.Acetyltransferase p=0.0396
CblA.beta.lactamase.Class.A p=0.0168
Tetracycline.Resistance.Ribosomal.Protection.Protein p=1.42e−14
CfxA.beta.lactamase.Class.A p=1.4e−07
Macrolide.MFS.Antibiotic.Efflux.Pump p=0.00371
Erm.23S.Ribosomal.RNA.Methyltransferase p=0.000708
CblA.beta.lactamase.Class.A p=0.0344
LnuAN2_Lincosamide.Nucleotidyltransferase p=0.0028
ANT.6 p=0.000912
MphB.macrolide.phosphotransferase p=0.0177
Unclassified.beta.lactamase.Class.C p=0.00382
SMR.Antibiotic.Efflux.Pump p=0.0266
Tetracycline.ABC.Transporter p=0.0091
AAC.6 p=0.0554
CMY.LAT.MOX.ACT.MIR.FOX.beta.lactamase.Class.C p=0.000152
ABC.Transporter p=1.6e−08
Pmr.phosphoethanolamine.transferase p=5.8e−09
ampC.type.beta.lactamase.Class.C p=8.26e−10
MFS.Antibiotic.Efflux.Pump p=1.24e−11
RND.Antibiotic.Efflux.Pump p=6.49e−09
RND.Antibiotic.Efflux.Pump p=0.00114
MFS.Antibiotic.Efflux.Pump p=0.00766
Unclassified.beta.lactamase.Class.A p=0.0523
ABC.Transporter p=0.00306
Pmr.phosphoethanolamine.transferase p=0.03
ampC.type.beta.lactamase.Class.C p=0.0341
ANT.3 p=0.0311
MphB.macrolide.phosphotransferase p=0.011
Tetracycline.Resistance.MFS.Efflux.Pump p=0.0335
ANT.6 p=0.0248
Tetracycline.MFS.Antibiotic.Efflux.Pump p=0.0169
Tetracycline.Resistance.Ribosomal.Protection.Protein p=0.00021
CfxA.beta.lactamase..Class.A. p=0.00265
Tetracycline.Resistance.MFS.Efflux.Pump p=0.0509
Tetracycline.MFS.Antibiotic.Efflux.Pump p=0.0387
Tetracycline.Resistance.Ribosomal.Protection.Protein p=0.00987
MphA.macrolide.phosphotransferase p=0.00368
APH.3 p=5.84e−05
Tetracycline.ABC.Transporter p=0.0148
SAT.4_Streptothricin.Acetyltransferase p=5.04e−17
AAC.6 p=0.0163
TetX p=0.00397
ANT.9 p=2.18e−18
ANT.6 p=7.47e−11
Unclassified.beta.lactamase.Class.A p=0.00913
CblA.beta.lactamase.Class.A p=0.0282
Erm.23S.Ribosomal.RNA.Methyltransferase p=0.0128
CMY.LAT.MOX.ACT.MIR.FOX.beta.lactamase.Class.C p=0.0268
LnuAN2_Lincosamide.Nucleotidyltransferase p=0.0216
SMR.Antibiotic.Efflux.Pump p=0.0235
ANT.3 p=0.0574
ANT.9 p=0.00228
Unclassified.beta.lactamase.Class.C p=0.0295
CfxA.beta.lactamase.Class.A p=<2e−16
ANT.3 p=0.0237
ANT.9 p=0.0602
Campylobacter.coli.chloramphenicol.acetyltransferase p=0.000376
APH.3 p=0.00907
Tetracycline.MFS.Antibiotic.Efflux.Pump p=0.00698
ANT.6 p=0.0013
Erm.23S.Ribosomal.RNA.Methyltransferase p=0.0222
CatA.chloramphenicol.acetyltransferase p=0.0189
CblA.beta.lactamase.Class.A p=0.00537
Tetracycline.Resistance.Ribosomal.Protection.Protein p=0.00652

Fig. S13: Travel duration has a small but statistically significant effect on AMR gene acquisition.

A The x-axis is the travel duration in days and the y-axis shows if a traveler acquired a AMR gene or not. Each point refers to one gene in one individual. Genes are included if they were not found in the pre-travel timepoint. Point color indicates travel region. The p-value and estimate from a generalized linear model fit to these data is given in the top right of the panel. **B** shows the difference between the bootstrapped travel duration distributions of the TRUE (gene acquired) and FALSE (gene not acquired) groups. The lines give the 95% confidence interval for the difference and the point gives the estimate. Source data is provided in the source data file.

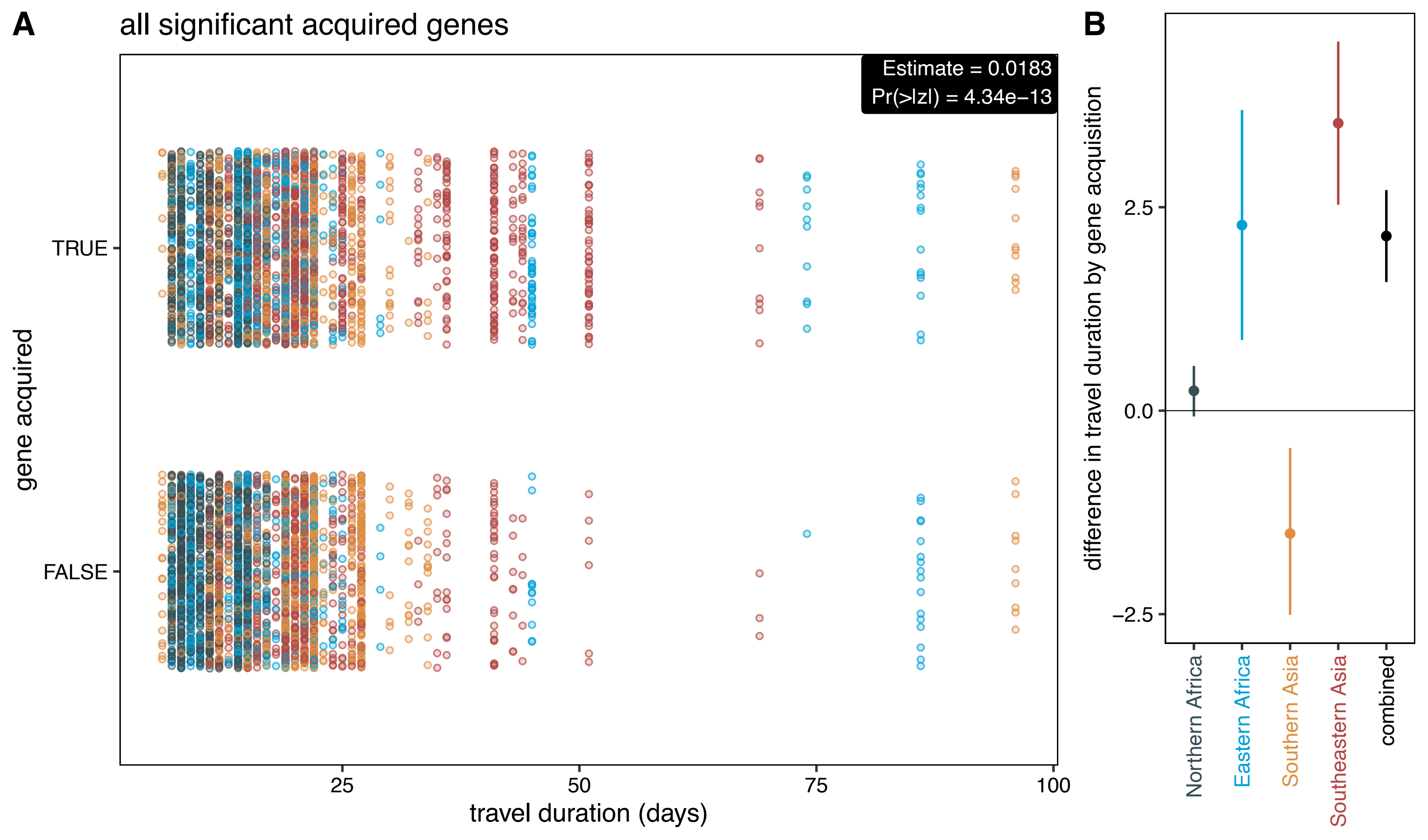
A
all significant acquired genes
Estimate = 0.0183
Pr(>|z|) = 4.34e−13
gene acquired
TRUE
FALSE
25
50
75
100
travel duration (days)
B
difference in travel duration by gene acquisition
2.5
0.0
−2.5
Northern Africa
Eastern Africa
Southern Asia
Southeastern Asia
combined

Fig. S14: Putative mobile genetic elements are more prevalent after travel.

A The left panel shows putative mobile genetic element counts detected in metagenomic assemblies normalized by the genomic content (megabases) in each assembly. Each point is a sample and the boxes are the medians with interquartile ranges for the pre-travel samples in blue and the post-travel samples in red. The p-value (paired Wilcoxon test) for the comparison is given at the top of the panel. The right panel shows the difference between the bootstrapped distributions of the post- and pre-travel samples. The red line gives the 95% confidence interval for the difference and the point gives the estimate. **B** The bottom panel shows the comparisons of AMR gene abundance before and after travel to the four subregions in this study. Points correspond to samples and boxes give the median and interquartile ranges. pre-travel is shown in blue and post-travel is shown in red. The p-values (fdr corrected paired Wilcoxon tests) for comparisons within region between the pre- and post-travel samples are shown above each comparison. The top panel shows the difference between the bootstrapped distributions of the post- and pre-travel samples. The red line gives the 95% confidence interval for the difference and the point gives the estimate. Source data for all panels is provided in the source data file.

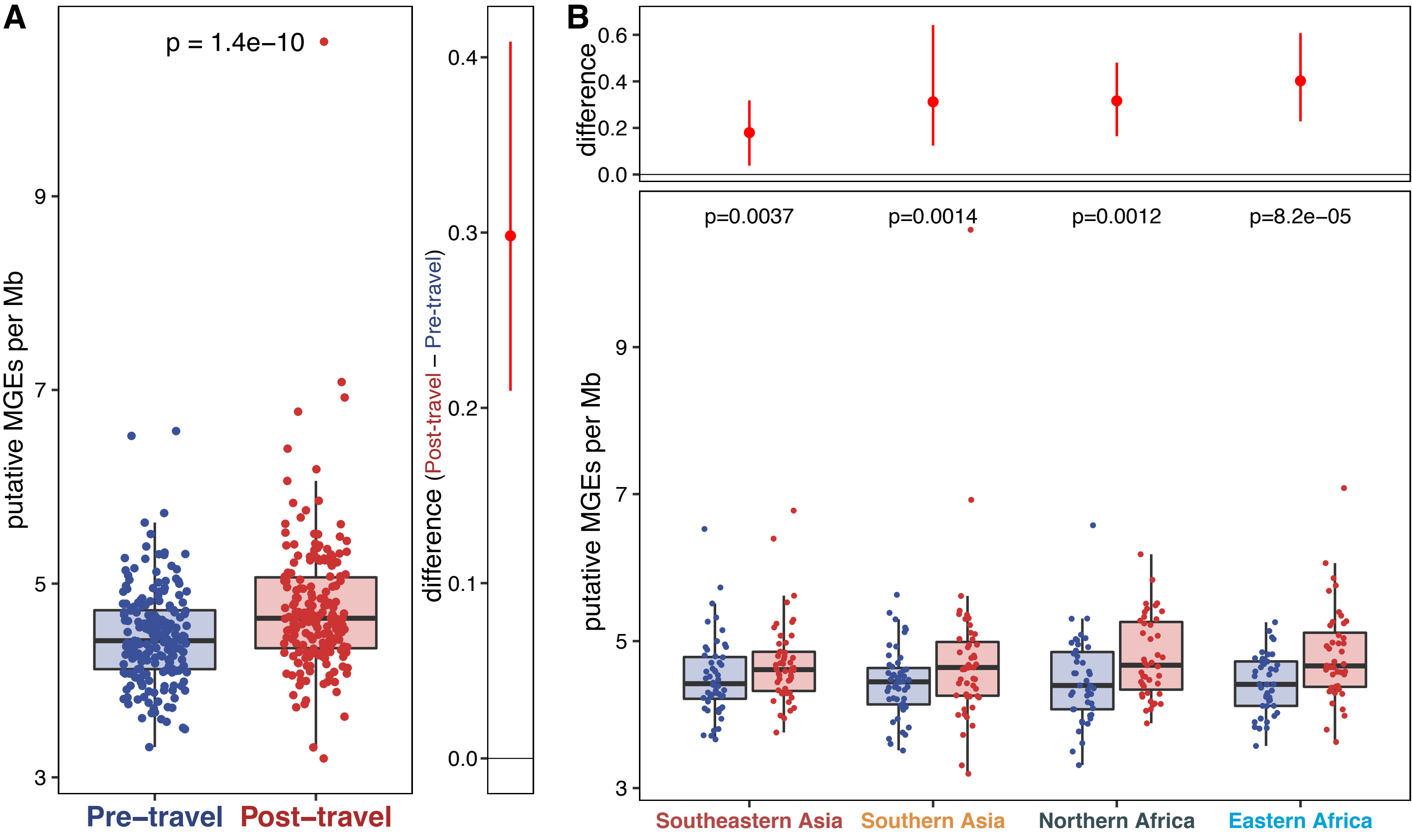
A
p = 1.4e−10
putative MGEs per Mb
3
5
7
9
Pre−travel
Post−travel
difference (Post-travel − Pre-travel)
0.0
0.1
0.2
0.3
0.4
B
difference
0.0
0.2
0.4
0.6
p=0.0037
p=0.0014
p=0.0012
p=8.2e−05
putative MGEs per Mb
Southeastern Asia
Southern Asia
Northern Africa
Eastern Africa

Fig. S15: Travel destination is not associated with putative mobile genetic element detection in post-travel sample metagenomic assemblies.

A Number of putative MGE elements per megabase detected in post-travel samples from the four travel subregions. The p-value for an ANOVA comparing the means for the four subregions is given in the top right of the panel. **B** shows the comparisons from panel A split by annotation type. The p-value for an ANOVA comparing the means for the four subregions for each annotation type is given in the top left of each panel. For all plots, points correspond to samples and boxes give the median and interquartile ranges. Source data for all panels is provided in the source data file.

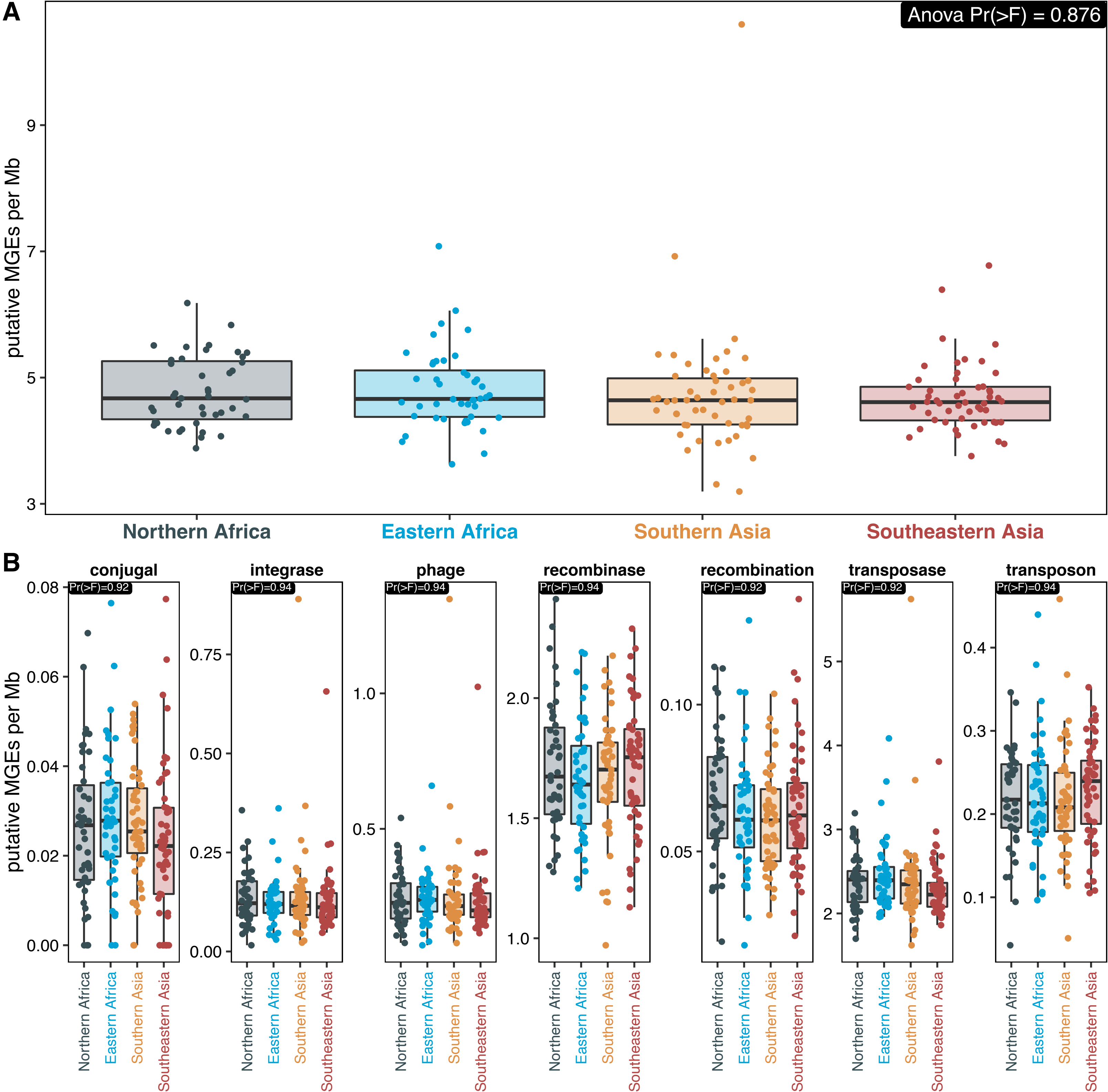

A
Anova Pr(>F) = 0.876
putative MGEs per Mb
3
5
7
9
Northern Africa
Eastern Africa
Southern Asia
Southeastern Asia
B
conjugal
integrase
phage
recombinase
recombination
transposase
transposon
Pr(>F)=0.92
Pr(>F)=0.94
Pr(>F)=0.94
Pr(>F)=0.94
Pr(>F)=0.92
Pr(>F)=0.92
Pr(>F)=0.94
putative MGEs per Mb
0.00
0.02
0.04
0.06
0.08
0.25
0.50
0.75
0.5
1.0
1.5
2.0
0.05
0.10
2
3
4
5
0.1
0.2
0.3
0.4
Northern Africa
Eastern Africa
Southern Asia
Southeastern Asia
